# *dnc-1/dynactin 1* Knockdown Disrupts Transport of Autophagosomes and Induces Motor Neuron Degeneration

**DOI:** 10.1371/journal.pone.0054511

**Published:** 2013-02-07

**Authors:** Kensuke Ikenaka, Kaori Kawai, Masahisa Katsuno, Zhe Huang, Yue-Mei Jiang, Yohei Iguchi, Kyogo Kobayashi, Tsubasa Kimata, Masahiro Waza, Fumiaki Tanaka, Ikue Mori, Gen Sobue

**Affiliations:** 1 Department of Neurology, Nagoya University Graduate School of Medicine, Nagoya, Japan; 2 Group of Molecular Neurobiology, Nagoya University Graduate School of Science, Nagoya, Japan; 3 Core Research for Evolutional Science and Technology (CREST), Japan Science and Technology Agency (JST), Saitama, Japan; Lousiana State University Health Sciences Center, United States of America

## Abstract

Amyotrophic lateral sclerosis (ALS) is a fatal neurodegenerative disease characterized by the progressive loss of motor neurons. We previously showed that the expression of dynactin 1, an axon motor protein regulating retrograde transport, is markedly reduced in spinal motor neurons of sporadic ALS patients, although the mechanisms by which decreased dynactin 1 levels cause neurodegeneration have yet to be elucidated. The accumulation of autophagosomes in degenerated motor neurons is another key pathological feature of sporadic ALS. Since autophagosomes are cargo of dynein/dynactin complexes and play a crucial role in the turnover of several organelles and proteins, we hypothesized that the quantitative loss of dynactin 1 disrupts the transport of autophagosomes and induces the degeneration of motor neuron. In the present study, we generated a *Caenorhabditis elegans* model in which the expression of DNC-1, the homolog of dynactin 1, is specifically knocked down in motor neurons. This model exhibited severe motor defects together with axonal and neuronal degeneration. We also observed impaired movement and increased number of autophagosomes in the degenerated neurons. Furthermore, the combination of rapamycin, an activator of autophagy, and trichostatin which facilitates axonal transport dramatically ameliorated the motor phenotype and axonal degeneration of this model. Thus, our results suggest that decreased expression of dynactin 1 induces motor neuron degeneration and that the transport of autophagosomes is a novel and substantial therapeutic target for motor neuron degeneration.

## Introduction

Autophagy is one of the major cellular systems that regulate protein degradation and organelle turnover in physiological and pathological conditions [1], and it is an essential quality control system for proteins in post-mitotic neurons that need to eliminate abnormal proteins and organelles for their proper function and survival [2], [3]. It is well known that the dysregulation of autophagy causes neurodegeneration [4], [5] and that the abnormal accumulation of autophagosomes is observed in several neurodegenerative diseases [6]–[9]. Particularly, intensified immunoreactivity for microtubule-associated protein 1 light chain 3 (LC3), which is a marker of autophagosome, is often observed in the spinal motor neurons of amyotrophic lateral sclerosis (ALS) patients [8], [10]. Electron microscopy of the motor neurons of ALS patients shows an increased number of autophagosomes surrounded by a double-membrane that contain sequestered cytoplasmic organelles, e.g., mitochondria [8]. Although these observations suggest the possibility that autophagy is upregulated to protect neurons from increased amounts of aggregated proteins and/or damaged organelles, it is also possible that the accumulation of autophagosomes due to dysregulated autophagy leads to neurodegeneration.

One possible mechanism for the accumulation of autophagosomes in degenerated neurons is the disruption of the cellular transport system, given that autophagosomes are cargo that moves bidirectionally along microtubules, which is powered by the kinesin family of motor proteins and dynein/dynactin complexes [11], [12]. We previously investigated the motor neuron-specific gene expression profile of sporadic ALS (SALS), which accounts for more than 90% of ALS, and found that the expression of dynactin 1, which is a key member of the dynactin family, is markedly decreased in the spinal motor neurons of SALS patients [9]. The decreased expression of dynactin 1 was also verified quantitatively using *in situ* hybridization analysis of tissues from SALS patients [13]. By contrast, the expression of other motor proteins including the kinesin family, which are responsible for anterograde transport and dyneins, which are responsible for retrograde transport was not significantly changed. Thus, we hypothesized that the decreased expression of dynactin 1 results in the disrupted transport of autophagosomes and thus attenuates the protective effects of autophagy against neurodegeneration.

Moreover, mutations of *DCTN1*, the gene encoding dynactin 1, are linked to familial lower motor neuron disease [14]. Several mutant *DCTN1* models exhibited motor dysfunction and pathological changes related to motor neuron disease [15], [16]. As seen in the motor neurons of SALS patients, mutant *DCTN1* mice exhibited a massive accumulation of membrane vesicles, including autophagosomes, in spinal motor neurons [16]. Although these findings suggest that impaired vesicular trafficking might cause the accumulation of vesicles, it remains unclear whether the transport of autophagosomes is actually impaired in the mutant *DCTN1* mice or whether the accumulation of autophagosomes plays a causative role in the pathogenesis of motor neuron degeneration.

The aim of the present study was to clarify the biological link between the quantitative loss of dynactin 1 and the disruption of autophagy. In particular, we examined whether the decreased levels of dynactin 1 induce motor neuron degeneration by hindering the transport of autophagosomes. To this end, we first examined the relationship between the decreased levels of dynactin 1, the accumulation of autophagosomes, and motor neuron degeneration in post-mortem tissues from SALS patients. Next, we created a *Caenorhabditis elegans (C. elegans)* model of the motor neuron-specific knockdown (KD) of *dnc-1*, the *C. elegans* homolog of human *DCTN1*, using small hairpin RNA (shRNA), and investigated whether the depletion of dynactin 1 impairs the transport of autophagosomes and thereby induces motor neuron degeneration. Using this model, we also explored therapeutic strategies targeting the transport of autophagosomes.

## Materials and Methods

### Protocols for the human samples

#### Ethics Statement

The collection of autopsied human tissues and their use for this study were approved by the Ethics Committee of Nagoya University Graduate School of Medicine, and written informed consent was obtained from the patients' next-of-kin. Experimental procedures involving human subjects were conducted in conformance with the principles expressed in the Declaration of Helsinki.

#### Immunohistochemistry

Six micrometer-thick sections from paraffin-embedded spinal cord sections from autopsied patients were prepared as described previously [17]: four patients with sporadic ALS (64.5±9.3 years-old; M:F = 2∶2) and four disease controls (73.5±5.4 years-old; M:F = 1∶3). The four control patients were diagnosed with progressive supranuclear palsy, multiple system atrophy, diffuse lewy body disease, and Parkinon's disease, respectively. The sections were first microwaved for 20 min in 50 mM citrate buffer, pH 6.0, then blocked with TNB blocking buffer (PerkinElmer, Hvidovre, Denmark) in Tris-buffered saline (pH 7.5) at room temperature for 30 min and incubated with a monoclonal antibody against LC3 (anti-LC3, 1∶40000; Medical & Biological Laboratories, Co., Nagoya, Japan) or dynactin 1 (anti-dynactin 1 H300; 1∶2000; Santa Cruz, Santa Cruz, CA, USA) overnight at 4°C. The subsequent procedures were carried out using the EnVision+Kit/HRP (DAB) (DAKO, Glostrup, Denmark) according to the manufacturer's protocol.

#### Quantitative assessment of immunohistochemistry

To assess LC3 immunoreactivity in spinal motor neurons, we included 4 ALS patients and 4 disease controls, and prepared 5 independent specimens from each subject. We counted about 200 motor neurons in ALS patients and about 400 neurons in control patients. The intensity of immunohistochemistry signals was quantified using a BZ-8000 fluorescent microscope and its software (BZ-Analyzer; Keyence, Osaka, Japan). Signal intensity was expressed as the individual intracellular cytoplasmic signal level (arbitrary absorbance units/mm^2^) per motor neuron by subtracting the mean background levels of 3 regions of interest in each section. The ventral spinal horn was defined as the gray matter ventral to the line through the central spinal canal perpendicular to the ventral spinal sulcus. To investigate the correlation between dynactin 1 and LC3 in individual motor neurons we used consecutive transverse spinal cord sections.

#### *In situ* hybridization

*In situ* hybridization for human tissue was performed as described previously [13]. We provide the detailed information in Materials and Methods S1.

#### Electron microscopy

Electron microscopy was performed on samples from 2 sporadic ALS patients (71 years-old male and 62 years-old female) and 2 disease control patients (68 years old male with multiple system atrophy and 60 years-old male with multiple system atrophy). Epoxy resin-embedded specimens of spinal anterior horn were cut into 70-nm ultrathin sections. Ultrathin sections were contrasted by staining with uranyl acetate and lead citrate. Sections were viewed with a JEM-1400EX electron microscope (JEOL, Tokyo, Japan) at 80 kV.

### Protocols for *C. elegans*

#### Ethics statement

All animal experiments were performed in accordance with the National Institute of Health Guide for the Care and Use of Laboratory Animals and were approved by the Nagoya University Animal Experiment Committee.

#### Culture of *C. elegans*

Standard methods were used to culture *C. elegans* on nematode growth medium (NGM) agar [18]. The animals were maintained at 20°C unless otherwise indicated. We provide the detailed information in Materials and Methods S1.

#### Constructs and *C. elegans* Strains

To generate transgenic *C. elegans*, plasmid DNA encoding acr2promotor::*shRNA*::*gfp* was injected into the gonads of young adult hermaphrodite N2 worms. We provide the detailed information for the shRNA vector and other co-injected proteins, i.e., SNB-1 and Lgg1, in Materials and Methods S1.

#### Whole Mount *in situ* Hybridization

Whole mount *in situ* hybridization of worms was performed as described previously [13], [19]. We provide the detailed information in Materials and Methods S1.

#### Phenotypic analysis of *C. elegans*

A lifespan assay was performed as described preciously [20], with some modifications. The Worms were allowed to lay eggs on a dish for 3–6 h to obtain synchronous progeny for the experiment. L4 worms were collected and transferred every 3 days to a fresh plate until the end of their reproductive life. The animals were scored as dead if they did not move when prodded with a platinum pick and did not show pharyngeal pumping.

A body bend assay, liquid thrashing assay, and video capture analysis were performed as locomotion assays. To examine the body bend frequency, exposed worms were transferred onto a fresh NGM plate and scored for the number of body bends performed in 3 min. A body bend was defined as a change in the direction of the part of the worm corresponding to the posterior bulb of the pharynx along the y-axis, assuming that the worm was traveling along the x-axis. We also performed a liquid thrashing assay as described previously [21], with some modifications. Briefly, the worms were put on a 6-cm NGM-coated plate with 3 ml of M9 media. The worms were allowed to settle for 30 s, their movements were captured by video for 30 s, and the number of thrashing movements was counted. We also analyzed the speed of movement using a video capture system as described previously [22]. Briefly, fully matured, adult worms were transferred individually to agar plates with no food. The movement of each worm was observed for 5 min and recorded using video equipment (Olympus, Tokyo, Japan) with a sampling rate of 30 frames/s. A computer-controlled microscope stage was automatically moved to center the worms in the visual field using a custom image analysis algorithm within the microscope's software package (MetaMorph; Universal Imaging Corp., West Chester, PA, USA). The midlines of the recorded worms were extracted from each image. All strains were randomized and scored on the same day.

#### Preparation of starved worms for the dietary restriction assay

All worms were synchronized by egg preparation [23]. The eggs were incubated at 20°C for 48 h in liquid medium. After 48 h, newly hatched worms were washed 3 times with distilled water, transferred to S basal medium without OP50, and incubated for 24 h. Worms were then picked randomly and used for the liquid thrashing assay.

#### Drug treatment

The worms were synchronized by egg preparation and incubated at 20°C for 24 h in liquid medium. They were then treated with rapamycin (LC Laboratories, Woburn, MA, USA) dissolved in ethanol at a final concentration of 10 or 100 μM, 3-methyladenine (3-MA) (SIGMA) dissolved in DMSO at a final concentration of 1 or 10 mM, or trichostatin A (TSA) (Tokyo Chemical Industry, Co., Tokyo, Japan) dissolved in DMSO at a final concentration of 1, 10, or 100 μM and incubated in liquid medium for 48 h. For controls (0 μM), ethanol or DMSO was added. Worms were then picked randomly and used for the liquid thrashing assay or microscopic analysis.

#### Primary neuronal cell cultures of nematodes

Primary neuronal cell cultures were prepared as described previously [24], with some modifications. In the present study, in order to obtain larger number of gravid animals, we cultured the worms in liquid medium (S basal medium with concentrated OP50) as described previously [25]. After incubation in liquid medium for 3 days, we performed egg isolation using lysis buffer (0.5 M NaOH/1% NaClO). Then we removed eggshell by enzymatic digestion using chitinase (SIGMA) and isolated embryonic cells were plated onto peanut lectin-coated glass bottom dishes (IWAKI, Tokyo, Japan).

#### Microscopic analysis

The worms were anesthetized by placing them in an 8-μl drop of levamisole (2 mM) on solidified pads of 2% agarose laid on slides. After coverslipping, the worms were examined under an LSM710 confocal microscope (Carl Zeiss Inc., Thornwood, NJ, USA). The regularity of SNB-1::DsRed localization/spacing was evaluated by measuring the distance between two neighboring fluorescent puncta of SNB-1::DsRed using ImageJ 1.43 software (National Institutes of Health). The axonal defasciculation index was measured as follows. The ventral nerve cord was divided into compartments consisting of two neighboring motor neurons. We counted the number of compartments with axonal defasciculation and divided it by the total number of compartments.

In vivo analysis of autophagosome mobility was performed as follows. Lgg1::DsRed worms were plated on an agar pad and observed using confocal microscopy. The red puncta, which represent autophagosomes, were observed for 1 min. The number of autophagosomes that moved within 1 min was divided by the total number of autophagosomes observed.

#### *In vitro* transport assay and image analysis

Time-lapse images were acquired at room temperature using a 63× oil-immersion objective (N.A 1.4) for live-cultured neuron analysis at 1–2 frames/s. The images were analyzed using Zen2008 (Zeiss) software. The run-length of Lgg-1 in primary motor neurons was measured by drawing a line over moving fluorescent puncta using Zen2008. Motile puncta were counted only if they moved continuously in the same direction for more than 2 frames and if their displacement was at least 2 μm. Some runs were terminated by a pause or reversal. To ensure the accuracy of the run-length measurements, we excluded moving puncta at the beginning and end of the movie. The velocity of Lgg-1 movements was obtained from the total distance traveled divided by the duration of the run.

#### *In vivo* transport assay and image analysis

Time-lapse images were acquired at room temperature using a 63× objective (N.A. 1.4) for live-worm analysis at 1 frame/s. The images were analyzed using Image J 1.43 software (National Institutes of Health). First, individual tracks of SNB-1 or Lgg1 movement were analyzed using the Multiple Kymograph plug-in, as described previously [26]. The velocity of the moving vesicles was tracked manually and their instantaneous velocity was extracted. To calculate the ratio of moving versus total vesicles, the number of vesicles that moved more than 2 μm during each time lapse period was divided with the total number of particles in each acquisition.

#### Electron microscopy of *C. elegans*

A conventional two-step fixation method was performed as described previously [27]. We provide the detail information in Materials and Methods S1.

#### Western Blot Analysis and Quantitative real-time PCR

Western blot analyses and quantitative real-time PCR were performed as described previously [28], [29]. We provide a detail description in Materials and Methods S1.

#### Statistical analysis

Statistical analyses were performed using StatView software version 5 (Hulinks, Tokyo, Japan). We used the Kaplan-Meier and log-rank test, Student's t-test, Mann-Whitney U test, and one-way analysis of the variance (ANOVA) with the Bonferroni or Dunnett's post-hoc test. Pearson's correlation coefficient was used to assess the correlation of variables.

## Results

### Dysregulated dynactin 1 expression and autophagy in degenerated spinal motor neurons in SALS patients

**Figure 1:**
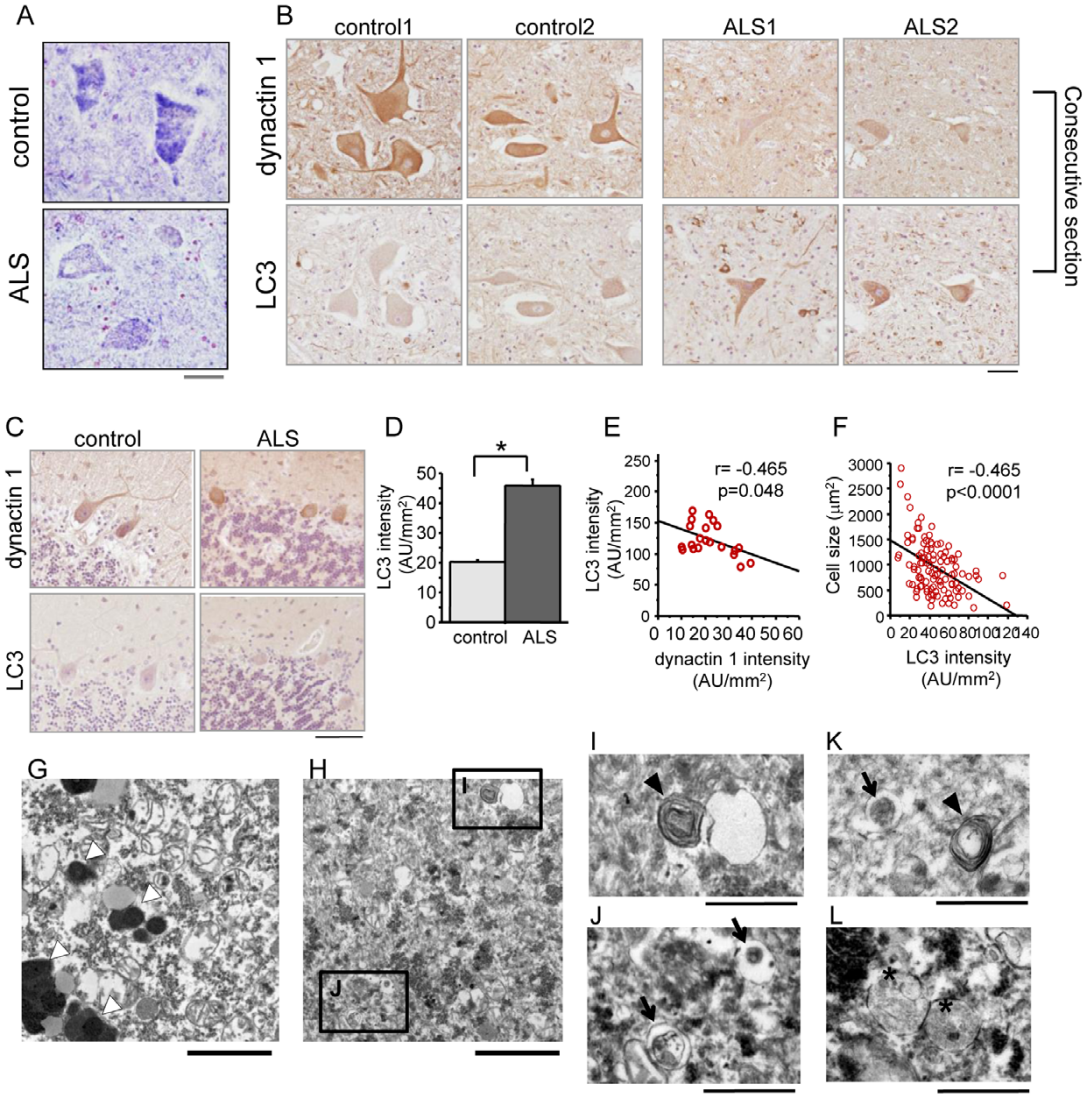
Dysregulated expression of dynactin 1 and the accumulation of autophagosomes in SALS patients. (*A*) Representative *in situ* hybridization for *DCTN1* in the spinal cords of control and ALS patients. (*B, C*) Representative immunohistochemistry for dynactin 1 and microtubule-associated protein 1 light chain 3 alpha (LC3) on consecutive spinal cord (*B*) and cerebellar (*C*) sections from control and ALS patients. (*D*) Quantification of the signal intensity of LC3 in anterior horn neurons of the spinal cord (n = 20 sections from 4 patients for each group). (*E*) Correlation between LC3 intensity and the expression of *DCTN1* in individual motor neurons from SALS patients (n = 12 consecutive sections from 3 SALS patients). (*F*) Correlation between the intensity of LC3 immunoreactivity and the size of motor neurons in SALS patients (n = 20 sections from 4 patients). (*G–L*) Electron microscopy images of spinal motor neurons. Representative lower magnification image of a motor neuron from a control patient (*G*) and lower (*H*) and higher magnification images (*I–L*) from SALS patients. The open arrowheads indicate lipofuscin. There were abundant autophagic vacuoles, e.g., multi-lamellar bodies (arrowheads in *I, K*), autophagosome-like double membrane vesicles (arrows in *K*, *J*), and autolysosomes (asterisks in *L*) in the motor neurons of SALS patients, but not of the control. Scale bar = 50 μm (*A–C*), 2 μm (*G, H*), or 1μm (*I–L*). Statistical analyses were performed using Student's t test (*p<0.0001) and Pearson's correlation coefficient in *E* and *F*. The error bars are S.E.M.

The expression of the *DCTN1* gene was markedly reduced in the spinal motor neurons of SALS patients, as reported previously [9], [13] (Fig. 1*A*). Recent studies indicate that the dysregulation of autophagy in motor neurons is a pivotal event in ALS [8], [10]; thus, we investigated the relationship between decreased dynactin 1 expression and autophagy in SALS. Immunohistochemistry using consecutive sections of autopsied human spinal cords revealed that LC3 immunoreactivity, a histological marker of autophagy, was increased in the motor neurons of SALS patients in which dynactin 1 expression was decreased (Fig. 1*B*). Conversely, there was no change in the immunoreactivity for dynactin 1 and LC3 in cerebellar Purkinje cells, which showed no degeneration (Fig. 1*C*). Quantitative analysis revealed that anti-LC3 immunoreactivity was significantly increased in the spinal motor neurons of SALS patients (p<0.0001) (Fig. 1*D*), and was inversely correlated with the decreased mRNA levels of *DCTN1* (Fig. 1*E*) and cell size (Fig. 1*F*) in the motor neurons of SALS patients, indicating that the dysregulation of autophagy is associated with the decreased expression of dynactin 1 in SALS. Electron microscopy of sections from the SALS and control patients (Fig. 1*G, H*) also revealed that there was an abundance of autophagic vacuoles, e.g., multi-lamellar bodies (arrowheads in Fig. 1*I, K*), autophagosome-like double membrane vesicles (arrows in Fig. 1*K, J*), and autolysosomes (asterisks in Fig. 1*L*) in the motor neurons of the SALS patients, which were scarcely observed in the control patients.

### Generation of the *dnc-1*-depleted *C. elegans* model

**Figure 2:**
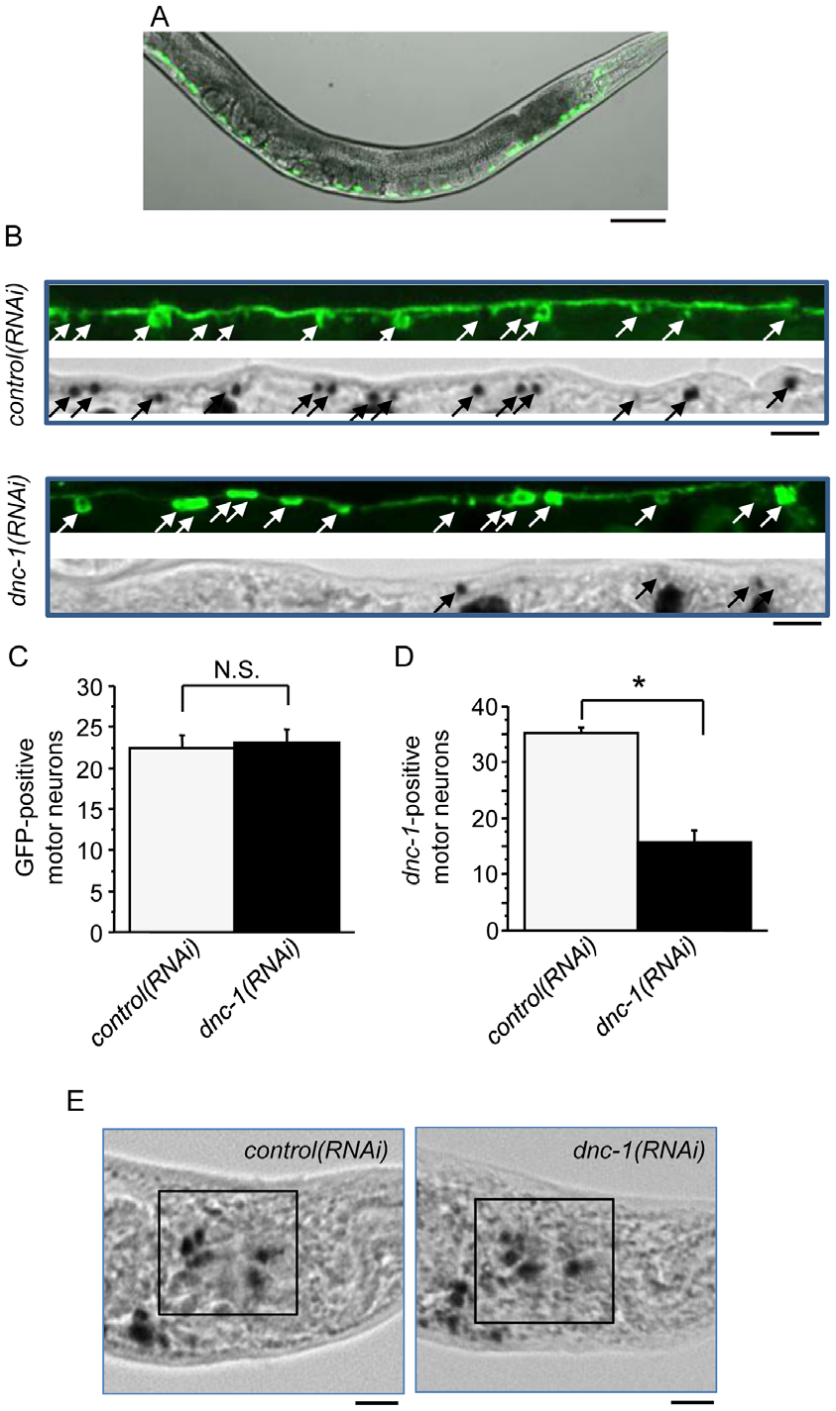
Creation of the motor neuron-specific *dnc-1-*KD *C. elegans* model. (*A*) Fluorescent visualization of ventral cholinergic motor neurons and their neurites in transgenic *C. elegans* worms expressing acr2p::*shRNA*::*gfp*. (*B*) Representative immunohistochemical staining of GFP and *in situ* hybridization against *dnc-1* in ventral cholinergic motor neurons and their neurites in the *control(RNAi)* and *dnc-1(RNAi)* worms. (*C*) The number of GFP-positive motor neurons (white arrows in *B*) was not significantly different between the *control(RNAi)* and *dnc-1(RNAi)* worms (n = 20 animals for each strain). (*D*) Conversely, the number of *dnc-1* mRNA-positive neurons (black arrows in *B*) was remarkably decreased in the *dnc-1(RNAi)* worms (n = 20 animals for each strain). (*E*) Representative images of *in situ* hybridization for *dnc-1* in the head neurons. Scale bars = 100 μm (*A*), 10 μm (*B*), and 20 μm (*E*). Statistical analyses were performed using Student's t test (*p<0.0001). The error bars are S.E.M.

To examine the relationship between the loss of dynactin 1, the accumulation of autophagosomes, and motor neuron degeneration, we created a *dnc-1*-KD *C. elegans* model by transfecting *C. elegans* with a plasmid expressing an shRNA and GFP under the control of the motor neuron-specific *acr2* promoter (*dnc-1(RNAi)*). In the transgenic worms, GFP was expressed diffusely in ventral motor neurons (Fig. 2*A*). We confirmed the effect of RNA interference on the level of endogenous *dnc-1* mRNA using whole mount *in situ* hybridization. In the *control(RNAi)* worms, *dnc-1* expression was not altered by *shRNA::GFP* expression (Fig. 2*B*). Conversely, in the *dnc-1(RNAi)* worms, motor neurons expressing *shRNA::GFP* exhibited reduced or no expression of *dnc-1* (Fig. 2*B*). As shown in Fig. 2*C*, approximately 22 neurons were GFP-positive both in the *control(RNAi)* and *dnc-1(RNAi)* worms. The number of *dnc-1*-positive motor neurons was decreased by approximately 20 (*control(RNAi)* worms, 35.3±3.8; *dnc-1(RNAi)* worms, 15.9±9.8), suggesting that *dnc-1* was successfully knocked down in almost all the GFP-positive cells (Fig. 2*C, D*). Moreover, *dnc-1* expression was not affected in the head sensory neurons of the *dnc-1(RNAi)* worms, confirming the specificity of the promoter (Fig. 2*E*). Taking these results into account, in the following experiments, we selected the *dnc-1(RNAi)* and *control(RNAi)* worms expressing GFP in more than 30 motor neurons to avoid the influence of knockdown efficiency on the experimental results.

### Motor dysfunction in motor neuron-specific *dnc-1-*KD *C. elegans*

**Figure 3:**
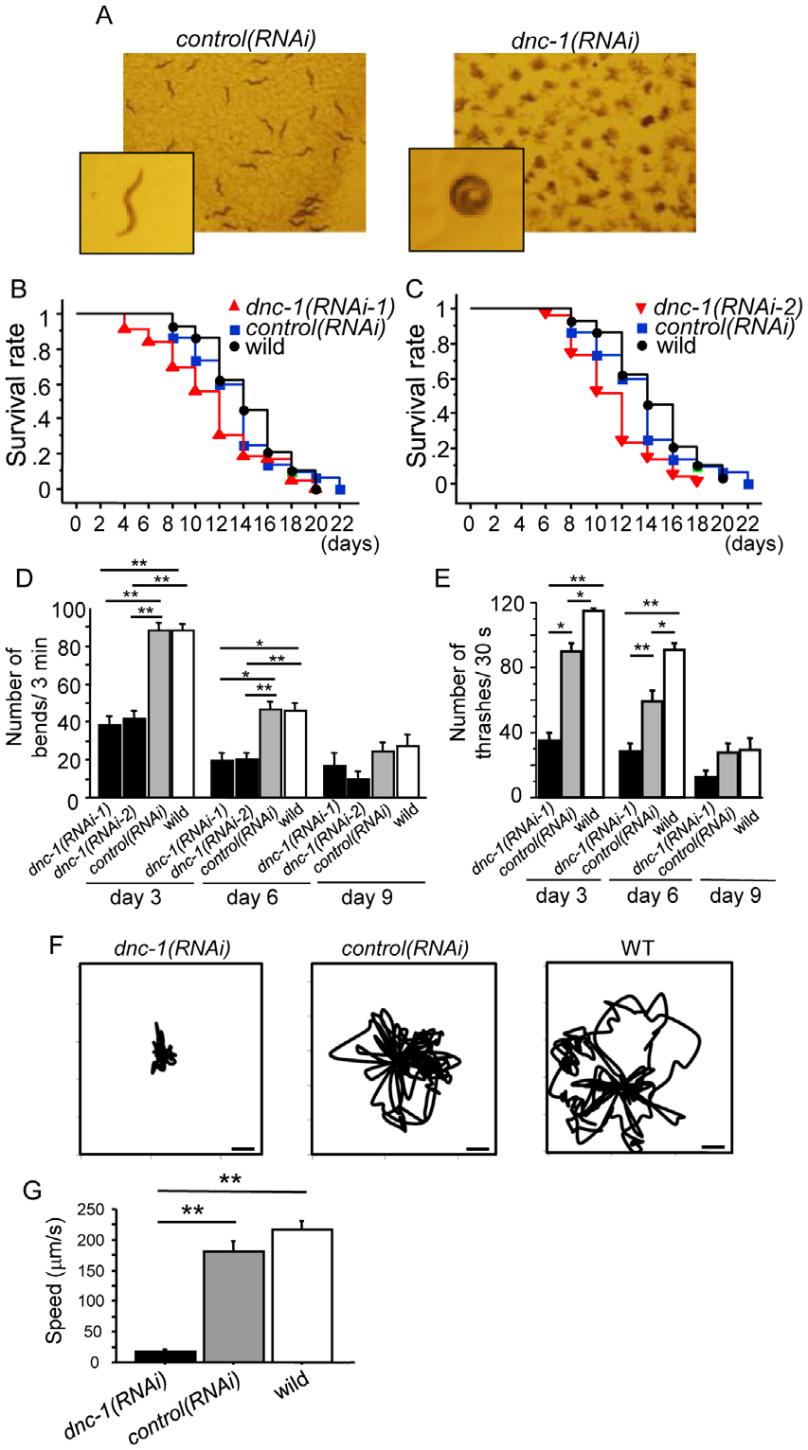
Motor dysfunction in the motor neuron-specific *dnc-1*-KD *C. elegans* model. (*A*) Stereoscopic microscopy showing the phenotypes of the *control(RNAi)* and *dnc-1(RNAi)* worms. (*B, C*) Survival curves of the transgenic worms (*dnc-1(RNAi-1)*, n = 90; *dnc-1(RNAi-2)*, n = 90; *control(RNAi)* n = 90; and wild-type n = 30). The same survival data of the *control(RNAi)*and wild-type worms were used in both graphs. Both *dnc-1(RNAi)* worms with different shRNA sequences (101, 2888) had significantly reduced life spans compared with the *control(RNAi)* worms (101: p = 0.005; 2888: p<0.0001; log-rank test). (*D*) The number of body bends associated with forward movement in 3 min. (*E*) The number of thrashing movements in liquid medium in 30 s. (*F, G*) The tracks (*F*) and average speed of the worms (*G*) analyzed by video capture at day 4. Scale bars in *F* = 100 μm. The error bars are S.E.M. (n = 30, 30, 40, and 40 for *dnc-1(RNAi-1)*, *dnc-1(RNAi-2)*, *control(RNAi)*, and wild-type, respectively, in *D*, *E*; and n = 6, 6, and 6 for *dnc-1(RNAi-1)*, *control(RNAi)*, and wild-type, respectively, in *G*). The statistical analyses in *C*, *D*, and *F* were performed by one-way ANOVA followed by the Bonferroni/Dunn post hoc test (*p<0.001 and **p<0.0001).

The *dnc-1(RNAi)* worms demonstrated uncoordinated locomotion (Fig. 3*A*), which is a phenotype observed in *C. elegans* mutant models of motor neuronal defects [30], [31]. Maturation of the worms resulted in the progressive aggravation of their uncoordinated locomotion, characterized by partial paralysis, slowed movement, and coiling. The feeding plate of the *dnc-1(RNAi)* worms appeared to be stagnated, as they only ate the food around themselves due to their decreased motility (Fig. 3*A*). As described in the Materials and Methods, we generated six lines of *dnc-1(RNAi)* worms: SBG7, 8, and 15 using shRNA1(101), and SBG20, 24, and 25 using shRNA2(2888). Survival analysis and body bend assays were performed using these six lines. Since these animals exhibited almost the same phenotype, SBG8 was employed for further analysis. Compared with the *control(RNAi)* worms, the *dnc-1(RNAi)* worms had a decreased life span (Fig. 3*B, C*) (11.4±4.4, 11.2±3.0, 13.4±4.0, and 14.3±3.3 days for *dnc-1(RNAi-1)*, *dnc-1(RNAi-2)*, *control(RNAi)*, and wild-type worms, respectively). *dnc-1(RNAi)* worms also exhibited significantly reduced bending and thrashing rates that declined with age (Fig. 3*D, E*). The thrashing speed of the *control(RNAi)* worms was slightly decreased compared with the wild-type worms, possibly due to the toxicity of GFP, as previously reported [32] (Fig. 3*E*). Although the toxicity of GFP was much less than that of *dnc-1* knockdown and not detectable in the bending assay, to exclude any effects of the fluorescent protein on our analysis, we compared the *dnc-1(RNAi)* worms with the *control(RNAi)* worms, both of which express GFP at similar levels, in all experiments. We also performed a video capture analysis to visualize the movement trace of each worm and measure its average speed (Fig. 3*F, G*). The movement speed was dramatically decreased in the *dnc-1(RNAi)* worms compared with the *control(RNAi)* worms at an early adult stage.

### Axonal degeneration is the early sign of neurodegeneration in the *dnc-1(RNAi)* worms

**Figure 4:**
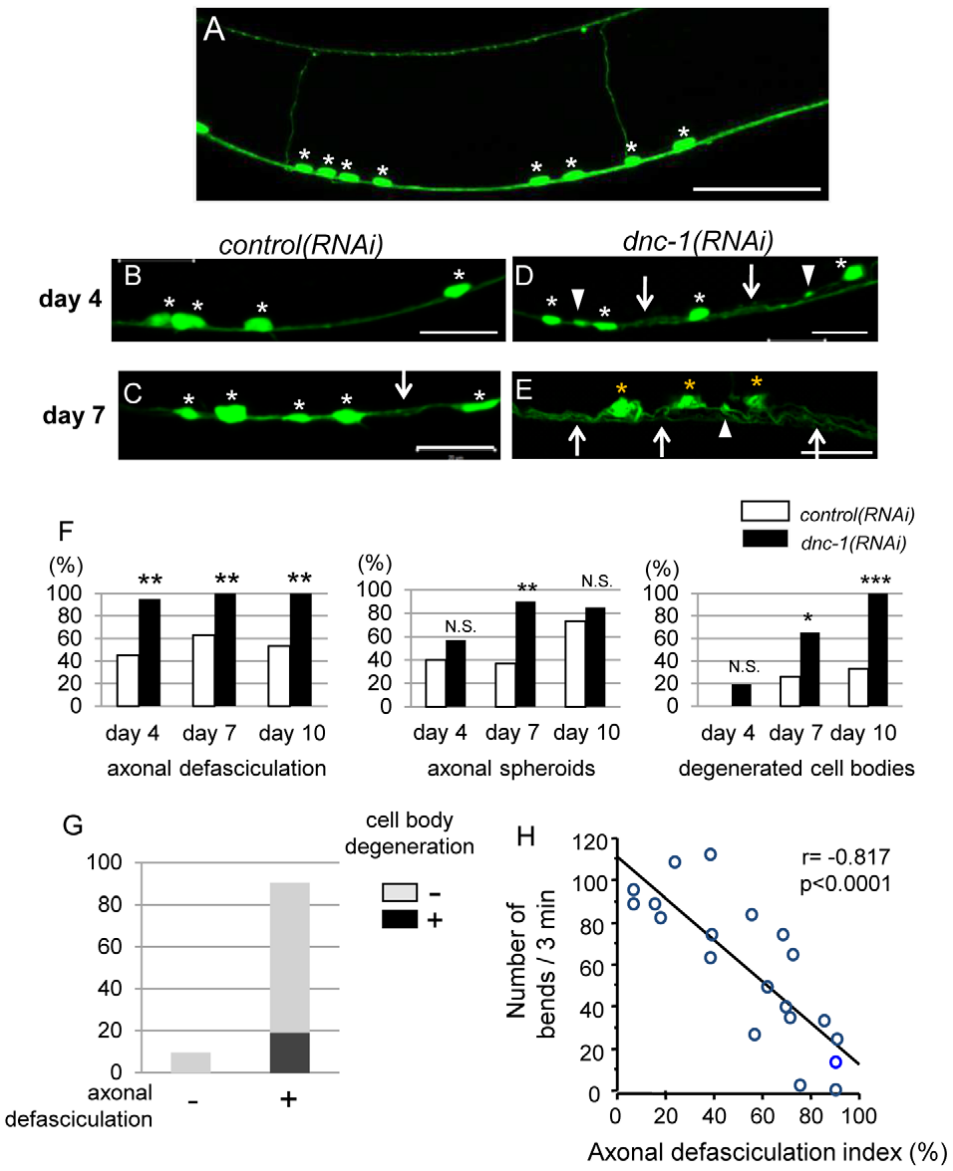
Morphological changes in ventral motor neurons. (*A*) Representative view of fluorescent GFP microscopic images of the ventral nerve cord in a *control(RNAi) C. elegans*. All of the motor neurons (white asterisks) were located in the ventral side of the worm. Axons from the motor neurons project within the ventral nerve cord or toward the dorsal side. (*B–E*) Representative view of the ventral nerve cord in the *control(RNAi)* worms (*B, C*) and *dnc-1(RNAi)* worms (*D, E*). The degenerated axons were defasciculated (arrows in *D, E*) and formed spheroids (arrowheads in *D, E*) in the *dnc-1(RNAi)* worms. Mild defasciculation was observed occasionally in the *control(RNAi)* worms (arrow in *C*). While the cell bodies of the motor neurons were regular and round in *control(RNAi)* and young adult *dnc-1(RNAi)* worms (white asterisks in *B–D*), abnormally shaped cell bodies (yellow asterisks in *E*) were observed only in the worms with severe axonal changes. (*F*) Semi-quantification of the abnormal morphological changes in the *control(RNAi)* and *dnc-1(RNAi)* worms. The percentage of worms with axonal defasciculation, axonal spheroids, or cell body degeneration on days 4, 7, and 10. (*G*) Population of *dnc-1(RNAi)* worms with and without cell body degeneration (black and gray boxes, respectively) on day 4. (*H*) Correlation between the axonal defasciculation index and locomotor function in the *dnc-1(RNAi)* worms. The axonal defasciculation index represents the degree of axonal defasciculation (its details are described in the Materials and Methods). Scale bars = 20 μm. The statistical analysis in *F* was performed using Fisher's exact probability test (*p<0.05, **p<0.001, and ***p<0.0001) and Pearson's correlation coefficient in *H*.

We then examined the morphological changes in the *dnc-1(RNAi)* worms using fluorescent microscopy. In normal worms, the ventral nerve cords were tightly fasciculated and the motor-neuron cell bodies (white asterisks in Fig. 4*A*) were round or ovoid. (Fig. 4*B, C*). By contrast, we found irregular shapes and defasciculation of the ventral nerve cord as well as axonal swellings, or spheroids, in the *dnc-1(RNAi)* worms at an early stage (Fig. 4*D*). At this early stage (4 days old), the cell bodies in the *dnc-1(RNAi)* worms seemed normal judging from their shape and structure (Fig. 4*D*). However, at a later adult stage (7 days old), axonal degeneration was exacerbated and morphological changes were also detected in the cell bodies (Fig. 4*E*). Axonal changes were occasionally observed in the *control(RNAi)* worms with aging, but they were less frequent and not as severe as in the *dnc-1(RNAi)* worms (Fig. 4*C*). Semi-quantification of the axonal and cell body changes showed that the axonal abnormalities were observed at day 4 and cell body deformation occurred at a later stage (Fig. 4*F*). Although some neurons exhibited an abnormal cell body shape at day 4, this change was only observed in the worms with axonal defasciculation (Fig. 4*G*), indicating that axonal degeneration occurs prior to cell body degeneration. Moreover, we also found that the severity of axonal defasciculation (i.e., the axonal defasciculation index) was correlated with locomotor dysfunction in the *dnc-1(RNAi)* worms (Fig. 4*H*). To clarify the time-course of the neuronal changes due to *dnc-1* depletion, we also examined the morphological change during the developmental stage. The acr2p::shRNA::GFP is not detectable before larval stage L1 (Fig. S1 *A–C*). Furthermore, even after GFP is expressed, there was no alteration in morphology or motor phenotype during the larval stage (from L1 to L4, post natal days 1 and 2) (Fig. S1 *C–E*). It was only after the worms became adult that the axonal degeneration and motor deficit appeared. Taken together, these findings suggest that the depletion of *dnc-1* induces the degeneration, rather than developmental defects, of motor neurons in *C. elegans*.

**Figure 5:**
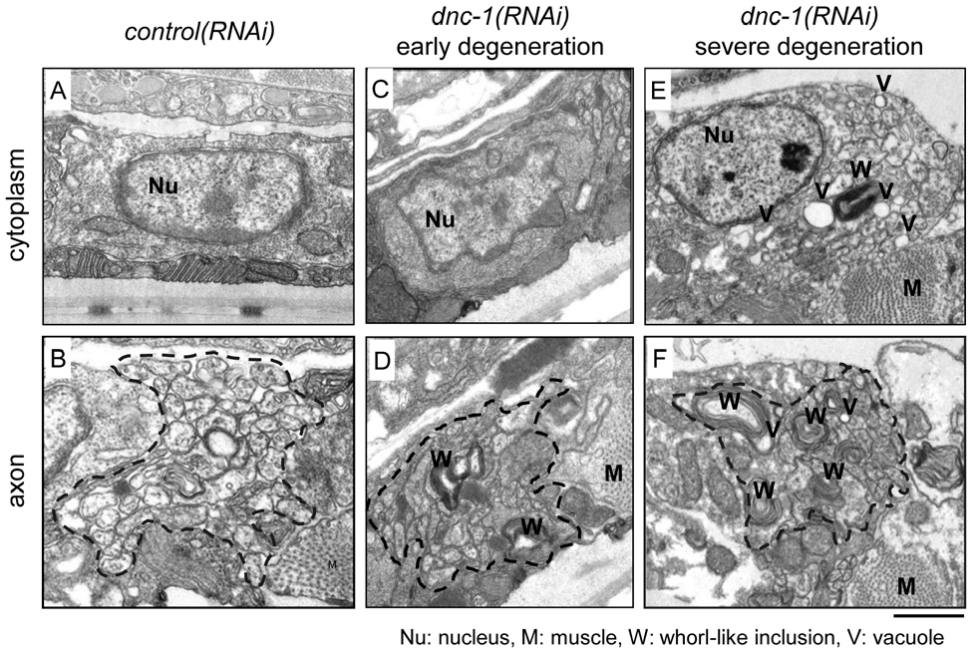
Ultrastructure of degenerating motor neurons. Electron microscopy of transverse sections of ventral motor neurons from the *control(RNAi)* (*A, B*) and *dnc-1(RNAi)* (*C–F*) worms. The dashed lines in *B*, *D*, and *F* denote the boundaries of the main bundle of axons. Each round-shaped component inside the dashed line is an axon. In the *dnc-1(RNAi)* worms, whorl-like inclusions (W) and vacuoles (V) were observed (*D–F*). In the worms with mild axonal degeneration (*D*), few morphological changes were observed in the cytoplasm (*C*); however, in the later stage with severe axonal degeneration (*F*), the cell bodies were also affected (*E*). Scale bars = 20 μm.

Further analysis via electron microscopy confirmed the axonal degeneration in the *dnc-1(RNAi)* worms (Fig. 5*C–F*). In the early degenerative stage, *dnc-1(RNAi)* worms first exhibited whorl like inclusions in axons with only a few morphological changes in their cell bodies (Fig. 5*C, D*) compared with *control(RNAi)* worms (Fig. 5*A, B*). In the later degenerative stage, strikingly abundant whorl-like inclusions and vacuoles, corresponding to degeneration and swelling of axons [21], were observed in axons and cell bodies (Fig. 5*E, F*).

### Axonal transport defect in the *dnc-1(RNAi)* worms

**Figure 6:**
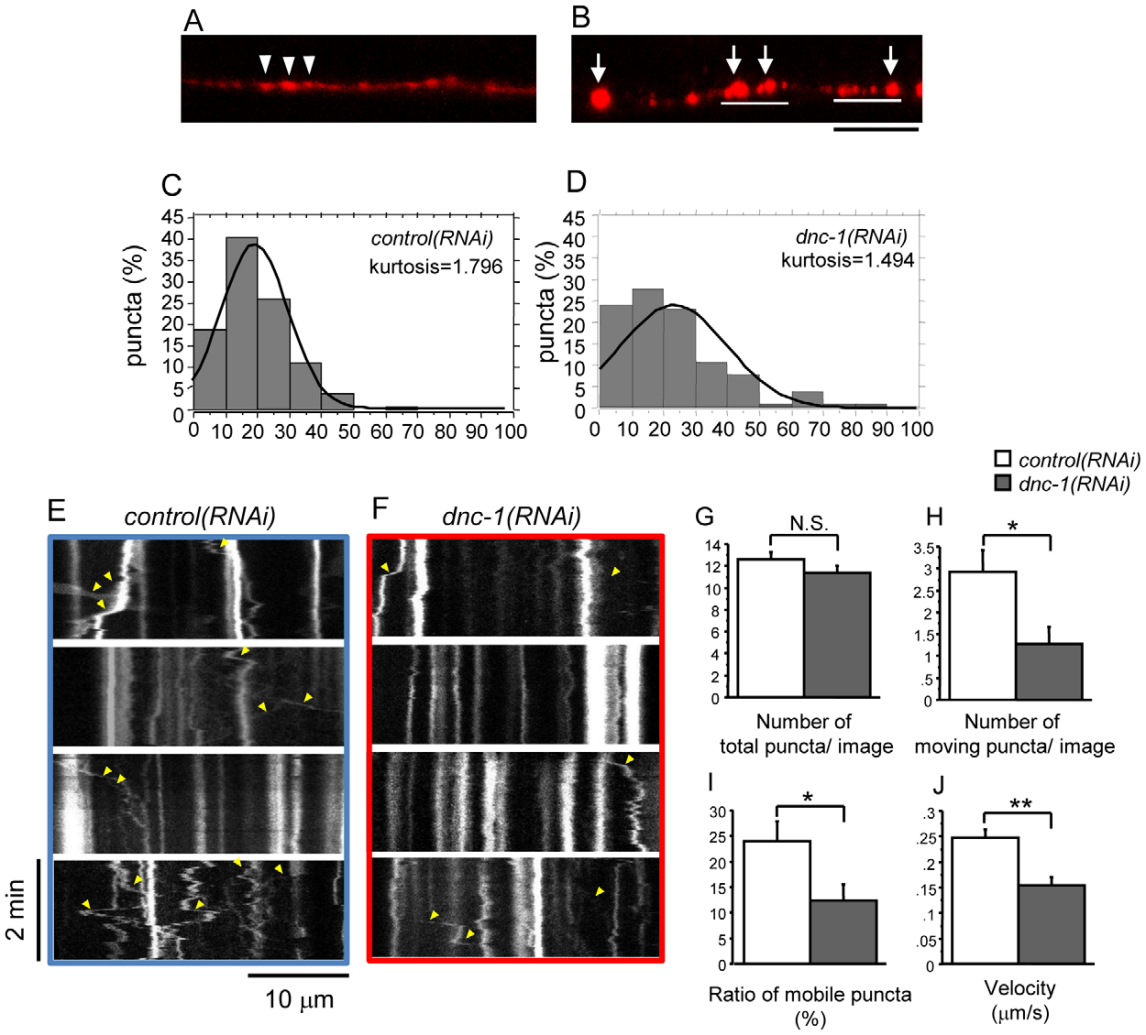
Defective axonal transport of synaptobrevin-1 in *dnc-1* *(* *RNAi* *)* *C. elegans*. (*A, B*) Expression patterns of DsRed-tagged synaptobrevin-1 (SNB-1) in the dorsal nerve cord. In the *control(RNAi)* worms, SNB-1 puncta (arrowheads) are regularly spaced with a uniform shape. In the *dnc-1(RNAi)* worms (B), they are irregularly spaced and abnormally accumulated (white bars) with occasional clumps. (*C, D*) Histograms of the distances between neighboring SNB-1 puncta. The average distance between puncta in the *control(RNAi)* (3.240±1.716 μm, n = 139) and *dnc-1(RNAi)* (3.855±2.764 μm, n = 104) worms was not significantly different (p = 0.996 by Student's t test), but the peak of the control histogram was higher than that of the *dnc-1(RNAi)* histogram, proving that the localization of SNB1 was irregular. (*E, F*) Representative kymographs of SNB-1::DsRed in the ventral nerve cord from the *control(RNAi)* (*E*) and *dnc-1(RNAi)* (*F*) worms derived from time-lapse imaging. Vertical lines represent stationary/docked SNB-1 puncta and oblique lines (labeled with yellow arrowheads) represent the tracks of moving SNB-1 puncta. The slope of this track is an indicator of velocity. (*G*) The number of SNB-1 puncta within a single image of kymograph was not different between the *control(RNAi)* and the *dnc-1(RNAi)* worms. (*H*) The mean velocities of SNB-1 puncta. (*I, J*) The quantitative analysis of mobile puncta. The number of puncta which moved more than 2 μm was counted (*I*). The ratio of moving puncta was calculated by dividing the number of moving puncta by the total number of SNB-1 puncta (*J*). A total of 20 time laps images were analyzed from each strains in *G*–*J*. Scale bar (black)  = 10 μm (*B*). Statistical analyses were performed using Student's t test (*p<0.05, **p<0.001, ***p<0.0001). Error bars are S.E.M.

Abnormalities in the localization and accumulation of synaptic vesicles were reported in a *C. elegans* model showing a defect in axonal transport [20]. To determine whether our *dnc-1(RNAi)* model exhibited defects in axonal transport, we used a fluorescently tagged synaptic vesicle marker composed of the *C. elegans* VAMP2/synaptobrevin protein fused to DsRed (SNB-1::DsRed), and examined the distribution of the dorsally located red puncta (Fig. 6*A*). In the dorsal nerve cord (the axons of the ventral motor neurons) of the *control(RNAi)* worms, SNB-1::DsRed puncta were regularly spaced, whereas the *dnc-1(RNAi)* worms exhibited a discontinuous and irregular distribution of the marker, including occasional clumps that may represent the accumulation of cargo proteins (Fig. 6*B*). Histograms of the distances between neighboring SNB-1 puncta displayed a broader curve in the *dnc-1(RNAi)* worms than in the *control(RNAi)* worms, suggesting some defect in axonal transport caused by the knockdown of *dnc-1* (Fig. 6*C, D*).

To demonstrate direct evidence of a defect in axonal transport in our transgenic worms, we monitored the movement of SNB-1 puncta by acquiring a series of time-lapse images. The resulting kymographs showed that puncta in the *dnc-1* KD worms were markedly static compared with those in the controls, confirming the disruption of axonal transport following the reduction of *dnc-1* in *C. elegans* (Fig. 6*E, F*, Movies S1, S2). To quantify the movement of SNB-1, we analyzed 20 kymographs from each strain. While there was no significant difference in the number of SNB-1 puncta between the *control(RNAi)* and *dnc-1(RNAi)* worms (Fig. 6*G*), the number of moving puncta (moving more than 2 μm) (Fig. 6*H*) and the ratio of moving puncta to total puncta (Fig. 6*I*) were significantly decreased in the *dnc-1(RNAi)* worms compared to the *control(RNAi)* worms (p = 0.028 and p = 0.014, respectively). The velocity of SNB-1 transport in the *dnc-1(RNAi)* worms was significantly lower than in the *control(RNAi)* worms (p<0.0001, Fig. 6*J*).

### Impaired transport and accumulation of autophagosomes in the *dnc-1(RNAi)* worms

**Figure 7:**
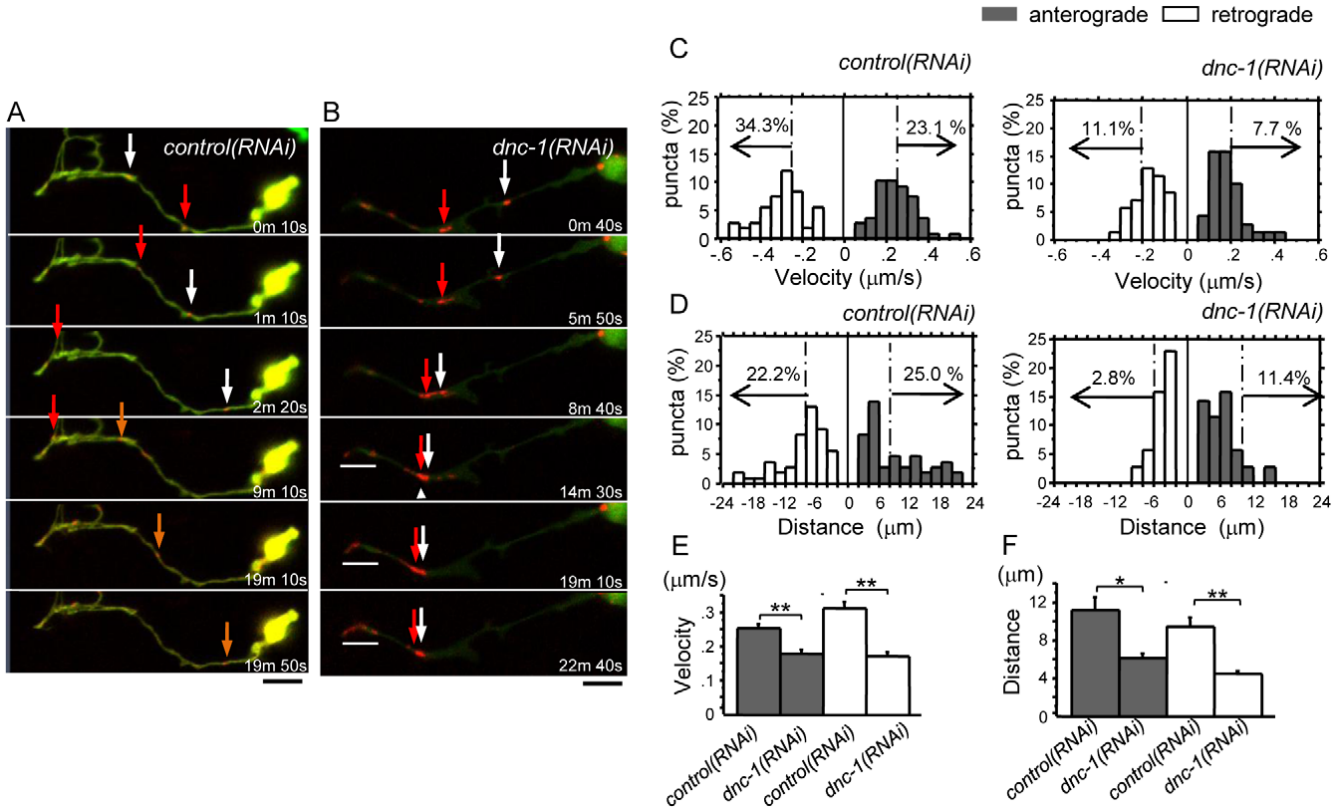
Impaired transport and abnormal accumulation of autophagosomes in the axons of *dnc-1* *(* *RNAi* *)* motor neurons. (*A, B*) Representative time-lapse images of autophagosome (DsRed-tagged Lgg1) transport in an axon (GFP-tagged shRNA; green) of a primary cultured motor neuron from the *control(RNAi)* (*A*) and *dnc-1(RNAi)* (*B*) worms. The autophagosomes were transported smoothly along the axon (arrows) of the *control(RNAi)* motor neuron (*A*). The autophagosome (arrows) was transported anterogradely, but was trapped where the axon was slightly narrowed (arrowhead) (*B*). There were also autophagosomes that accumulated in the distal part of the axon (*B*, bar). (*C*) Histograms of Lgg1::DsRed velocity in the retrograde (white bars) and anterograde (black bars) directions in neurons from the *control(RNAi)* and *dnc-1(RNAi)* worms. (*D*) Histograms of Lgg1::DsRed run-length in the *control(RNAi)* and *dnc-1(RNAi)* neurons. (*E, F*) Mean velocity (*E*) and run-length (*F*) of autophagosomes (n = 70 vesicles for each strain) in *control(RNAi)* and *dnc-1(RNAi)* neurons. Scale bar = 5 μm (A and B). The statistical analyses in *E* and *F* were performed using the Mann-Whitney U test (*p<0.05 and **p<0.0001). The error bars are S.E.M.

We next investigated the effects of *dnc-1* depletion on autophagy in *C. elegans.* Autophagosomes are cargo that moves bidirectionally along microtubules, powered by the kinesin family of motor proteins and dynein/dynactin complexes [11], [12]. Altered autophagy has been observed in several neurodegenerative models, including the mutant *DCTN1* mouse model [9], [16], [33], [34]. However, little is known about the relationship between the decreased levels of dynactin 1 and the alteration of autophagy. To clarify the effect of quantitative loss of DNC-1/dynactin 1 in the transport of autophagosomes, we performed live-cell imaging analyses of autophagosome transport in the axons of primary cultured motor neurons from the *dnc-1(RNAi)* and *control(RNAi)* worms that co-expressed DsRed-tagged Lgg1/ATG8, which is associated with the autophagic membrane, in ventral motor neurons under the control of the *acr2* promoter (Mizushima et al. [35]. This marker of autophagosomes is expressed diffusely in the ventral motor neurons (Fig. S2 *A*) and forms distinct puncta when autophagosomes are formed (Fig. S2 *B*) [36]. In the *control(RNAi)* neurons, the fluorescent Lgg1 vesicles moved toward and away from the cell body, suggesting that these vesicles are powered by anterograde and retrograde motors (Fig. 7*A*, Movie S3). By contrast, in the *dnc-1(RNAi)* worms, the autophagosomes were easily trapped where the axon was tight or curved, or at spheroids (Fig. 7*B*, Movie S4). This phenomenon was followed by the accumulation of autophagosomes distal to the trapped sites. Histograms showing the distribution of the velocity and distance of autophagosome movement demonstrated a significant loss of fast- and long-moving vesicles in the *dnc-1(RNAi)* cells compared with the *control(RNAi)* cells (Fig. 7*C, D*). The mean velocity and movement distance (run-length) were significantly decreased in the anterograde and retrograde directions in the *dnc-1(RNAi)* neurons (p<0.0001,  = 0.0001; velocity of anterograde, retrograde movements, respectively, and p = 0.0045, <0.0001; run-length of anterograde, retrograde movements, respectively) (Fig. 7*E, F*).

**Figure 8:**
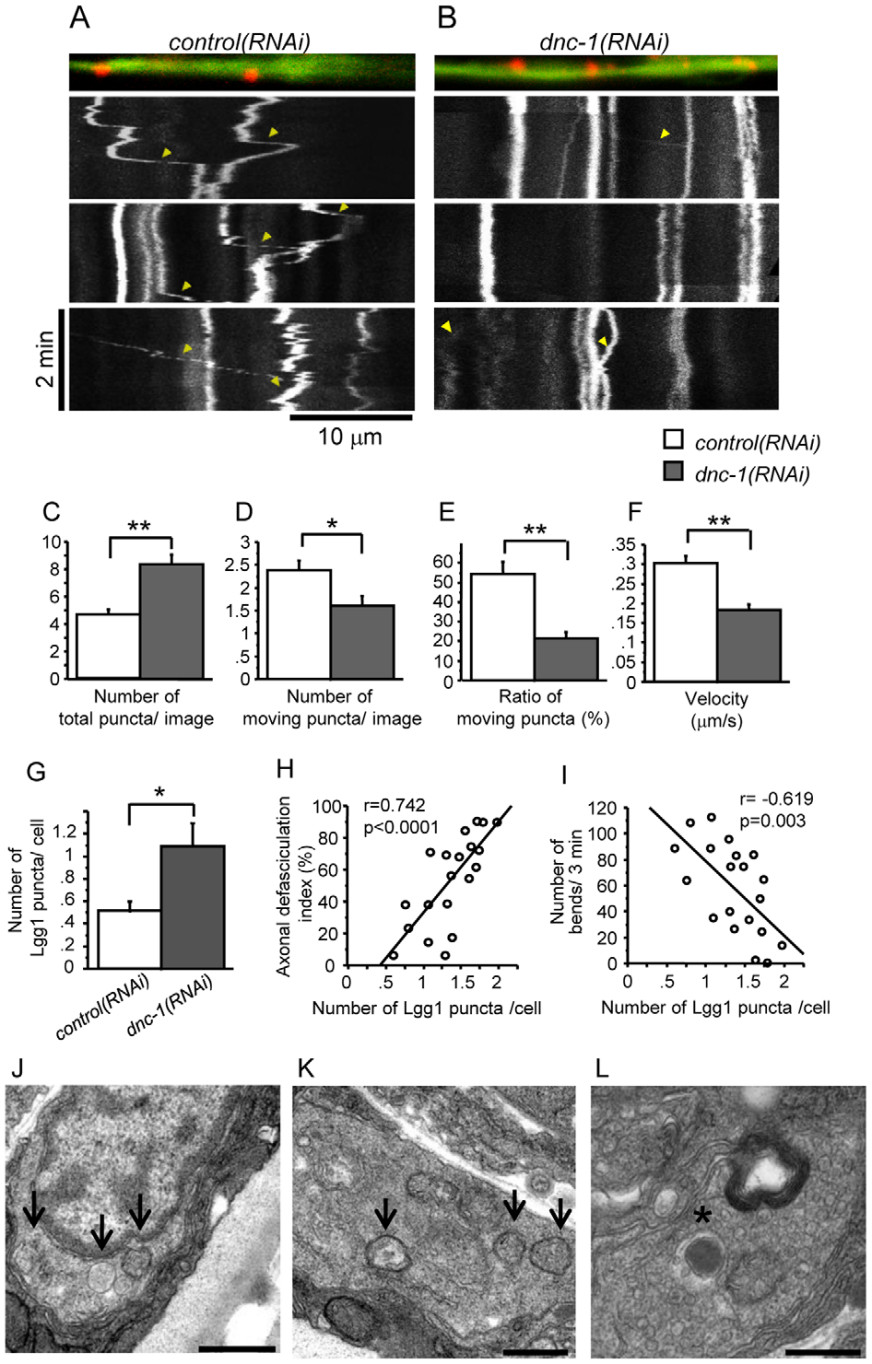
Accumulation of autophagosomes and motor neuron degeneration in the *dnc-1* *(* *RNAi* *)* worms. (*A, B*) Representative kymographs of Lgg1::DsRed in the ventral nerve cord from the *control(RNAi* (*A*) and *dnc-1(RNAi)* (*B*) worms derived from time-lapse images. Vertical lines represent stationary/docked Lgg1 puncta, while the oblique lines (labeled with arrowheads) represent the tracks of moving Lgg1 puncta. The slope of this track is an indicator of velocity. (*C*) The number of Lgg1 puncta within a single kymograph image. (*D, E*) Quantitative analyses of the mobility of puncta. The number of puncta that moved more than 2 μm was counted (*D*). The ratio of moving puncta was calculated by dividing the number of moving puncta by the total number of puncta (*E*). (*F*) The mean velocities of Lgg1 puncta. A total of 20 time-lapse images were analyzed for each strain in *C–F*. (*G*) The number of Lgg1 puncta was increased in the *dnc-1(RNAi)* worms compared with the *control(RNAi)* worms (n = 15 for each group). (*H, I*) Accumulation of autophagosomes in the *dnc-1(RNAi)* worms was correlated with the severity of axonal defasciculation (*H*) and locomotor function (*I*) (n = 20 for each analysis). (*J–L*) Ultrastructural images of ventral motor neurons from the *dnc-1(RNAi)* worms. Aberrant membranous vesicles including degenerated mitochondria were observed in the cytoplasm (*J*) and axons (*K*) (arrows). Autophagosome-like, double membrane vesicles (asterisk in *L*) were also found in the axons of the *dnc-1(RNAi)* worms (*L*). Scale bar = 500 nm (A–C) or 10 μm (D). Statistical analyses were performed using Student's t test (*p<0.05 and **p<0.0001) and Pearson's correlation coefficient in *H* and *I*. The error bars are S.E.M.

Next, we performed kymograph analysis of Lgg1::DsRed using *in vivo* time-lapse images (Fig. 8*A, B*, Movie S5, S6). Although the total number of Lgg1 puncta was significantly increased (p<0.0001) (Fig. 8*C*), the number (Fig. 8*D*) and the ratio of moving puncta (Fig. 8*E*) were significantly decreased in the *dnc-1(RNAi)* worms compared with the *control(RNAi)* worms (p = 0.013 and p<0.0001, respectively). The velocity of Lgg1 movement was also significantly decreased in the *dnc-1(RNAi)* worms (p<0.0001) (Fig. 8*F*). These results indicated that the *dnc-1* depletion resulted in the accumulation of untransported autophagosomes in the motor neurons.

We then investigated whether the accumulation of autophagosomes is related to the motor neuron degeneration. In the ventral nerve cord of the *dnc-1(RNAi)* worms, the number of Lgg1 puncta was significantly increased in comparison with the *control(RNAi)* worms (p = 0.019) (Fig. 8*G*), and the accumulation of autophagosomes was correlated with the axonal defasciculation index and locomotor function (Fig. 8*H, I*). We also explored the localization of Lgg1::DsRed in the distal ascending axon and observed Lgg1::DsRed accumulation in axonal spheroids (Fig. S2 *C*), which is consistent with a previous report showing the abnormal accumulation of disorganized organelles and autophagosomes in axonal spheroids [37]. Electron microscopy showed that the accumulation of vesicular structures, including autophagosome-like vesicles and mitochondria, was observed in the proximal axons or cytoplasm of the *dnc-1(RNAi)* worms, although such accumulations were detected rarely in the axons of the *control(RNAi)* neurons (Fig. 8*J–L*).

**Figure 9:**
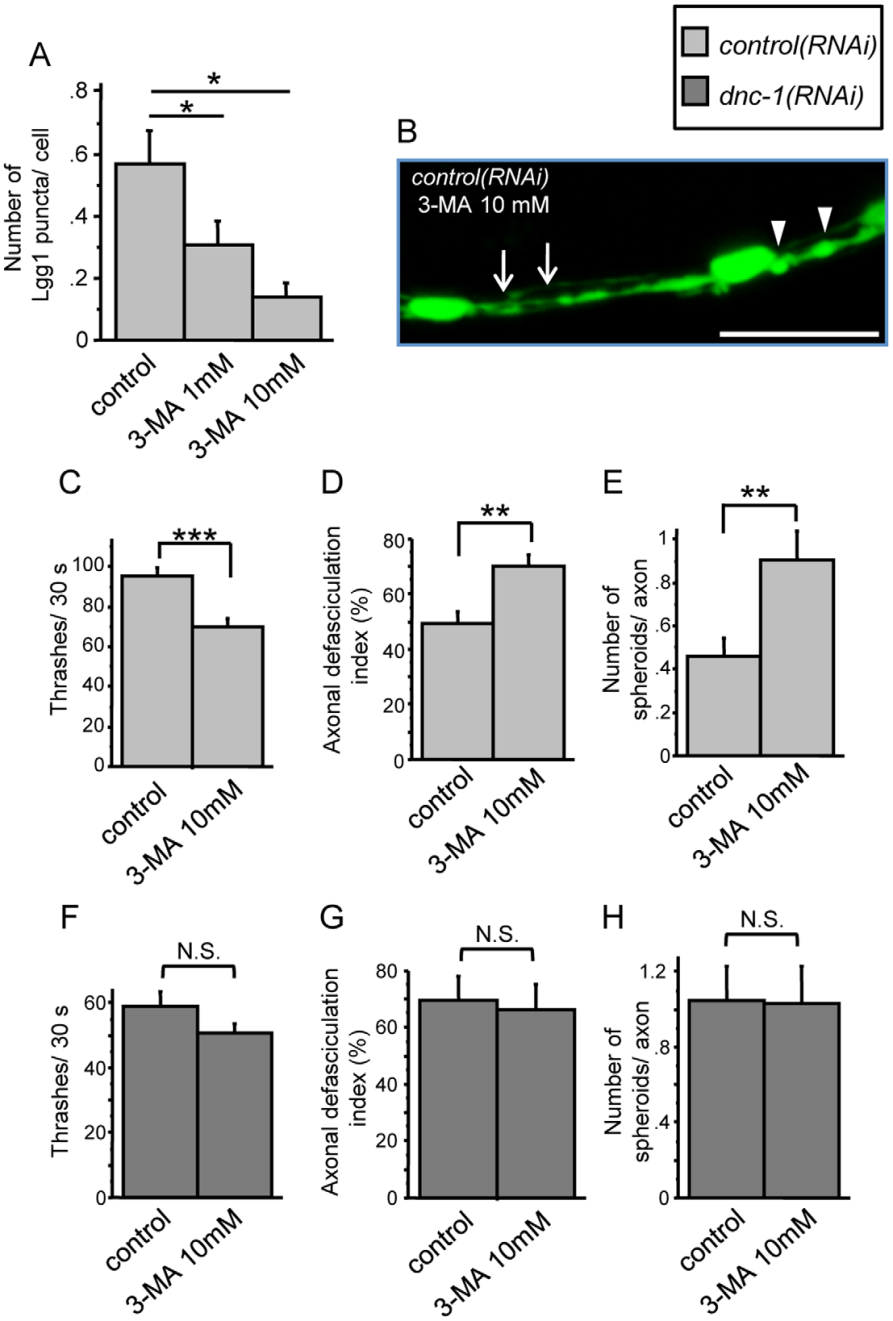
Dysfunction of autophagy causes axonal degeneration. (*A*) Treatment with 3-MA decreased the number of autophagosomes in the ventral nerve cord in a dose dependent manner (n = 15 for each group). (*B–E*) The effects of 3-MA on the locomotor function (*C*) and axonal morphology (*B, D,* and *E*) of the *control(RNAi)* worms. Treatment with 3-MA increased axonal defasciculation (arrows in *B* and the graph in *D*) and the number of axonal spheroids (arrowheads in *B* and the graph in *E*) (n = 15 for each group). (*F–H*) The effects of 3-MA on the locomotor function (*F*) and axonal morphology (*G, H*) of the *dnc-1(RNAi)* worms (n = 15 for each group). Scale bar = 10 μm. Statistical analyses were performed using Dunnett's post hoc test (*A*) and Student's t test (B, *D*, and *E*) (*p<0.05, **p<0.001, and ***p<0.0001). The error bars are S.E.M.

We then treated the *control(RNAi)* worms with 3-MA, which inhibits the formation of autophagosomes (Fig. 9*A*). These worms showed the locomotory defects and axonal degeneration observed in the *dnc-1(RNAi)* worms, suggesting that the disrupted autophagy system is sufficient to cause the motor neuronal degeneration in this model (Fig. 9*B–E*). On the other hand, when we treated the *dnc-1(RNAi)* worms with 3-MA, worms did not exhibit a substantial change in the motor function or in the axonal integrity (Fig. 9*F–H*).

### Starvation dramatically attenuates the motor deficits in the *dnc-1(RNAi)* worms by facilitating the axonal transport of autophagosomes

Autophagy is known to be activated by rapamycin, a specific inhibitor of the mTOR pathway [38]. Starvation is also a strong activator of autophagy; however, it also has other effects, e.g., activation of the mitogen-activated protein kinase (MAPK) pathway [39], stimulation of tubulin acetylation [40], and induction of sirtuin [41]. Both treatments have been used widely in many species, e.g., *Drosophila*, mouse, and *C. elegans*, to activate autophagy [42]–[44].

**Figure 10:**
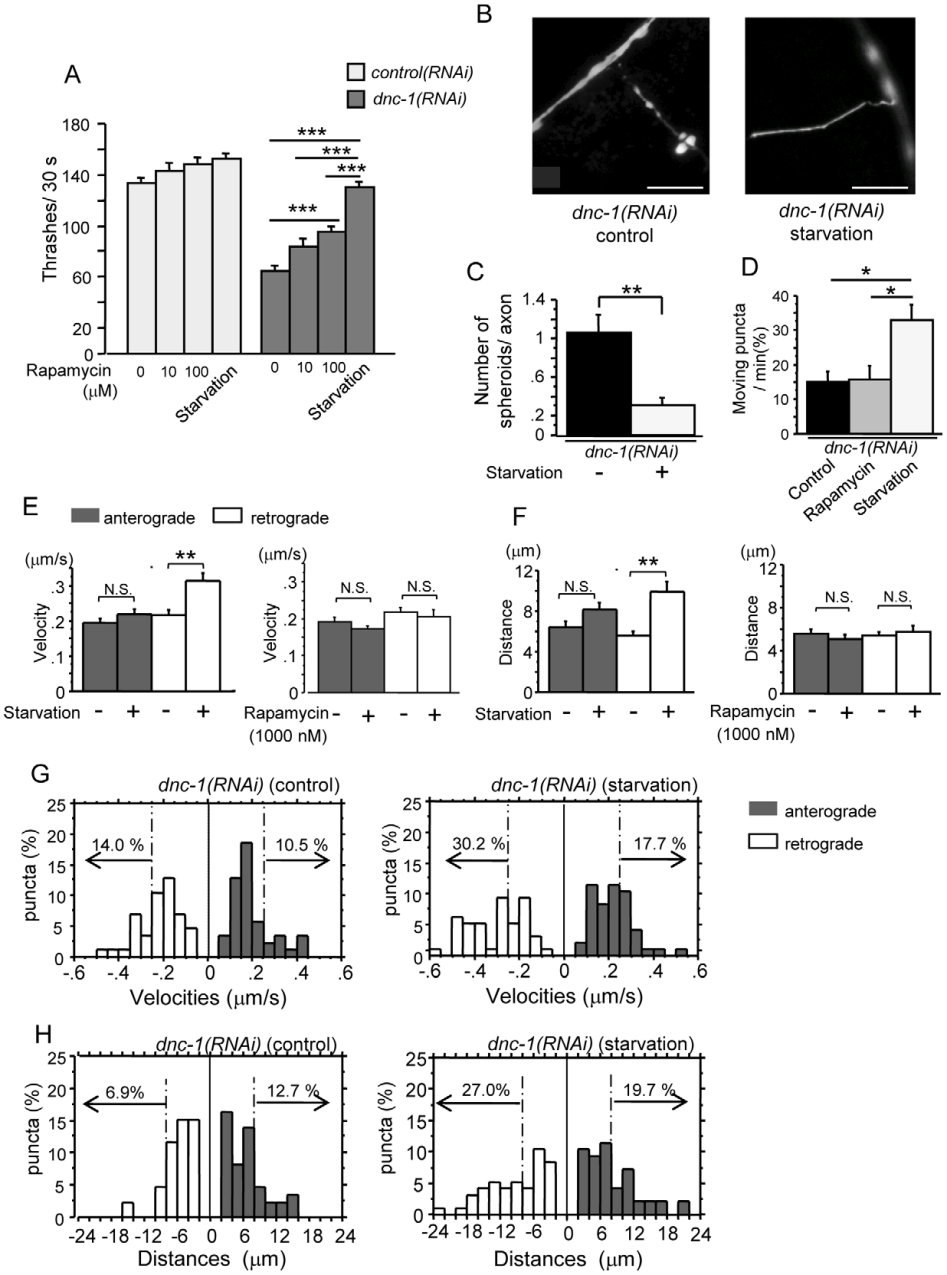
Starvation stimulates the retrograde transport of autophagosomes and attenuates axonal degeneration in the *dnc-1* *(* *RNAi* *)* worms. (*A*) Effect of rapamycin and starvation on locomotor function in the *control(RNAi)* and *dnc-1(RNAi)* worms (n = 50 for each group). (*B*) Fluorescent microscopy showing the morphological changes in axons after starvation in the *dnc-1(RNAi)* worms. (*C*) The number of axonal spheroids per transverse axon section in the *dnc-1(RNAi)* worms with or without starvation. (n = 15 animals for each treatment). (*D*) Effect of rapamycin (100 μM) and starvation on autophagosome mobility in the *dnc-1(RNAi)* worms. (n = 15 animals for each treatment). (*E, F*) Effect of rapamycin (100 μM) and starvation on the mean velocity (*E*) and run-length (*F*) of autophagosomes (black bars: anterograde transport; white bars: retrograde transport) (n = 70 vesicles for each treatment). (*G, H*) Histograms of Lgg1::DsRed velocity (*F*) and run-length (*G*) in the anterograde (black bars) and retrograde (white bars) direction in primary motor neurons from the *dnc-1(RNAi)* worms cultured with normal (control) and serum-free (starvation) medium. Scale bars = 5 μm. Statistical analyses were performed by one-way ANOVA followed by the Bonferroni/Dunn post hoc test (*A*) and Dunnett's post hoc test (*D*). Student's t test (*C*) and Mann-Whitney test (*E, F*) were used for two-group comparison (*p<0.05, **p<0.001, and ***p<0.0001). The error bars are S.E.M.

To study the effects of autophagy activators on axonal degeneration in *C. elegans*, we treated the *control(RNAi)* and *dnc-1(RNAi)* worms with rapamycin or starved them by food restriction, and investigated the changes in motor function via the liquid thrashing assay. Rapamycin and starvation are known to extend lifespan of *C. elegans* [42], [45]. In the present study, we found that neither rapamycin nor starvation significantly altered the motor function of the *control(RNAi)* worms (Fig. 10*A*). In the *dnc-1(RNAi)* worms, rapamycin ameliorated the thrashing activity in a dose-dependent manner, although it showed only a limited effect even at the most effective dose (Fig. 10*A*). In contrast, starvation completely ameliorated the motor dysfunction of the *dnc-1(RNAi)* worms without affecting the efficiency of *dnc-1* knockdown (Fig. 10*A*, Fig. S3 *A–C*). The formation of axonal spheroids was also significantly suppressed by starvation (p = 0.001) (Fig. 10*B, C*). Given the differential effects of rapamycin and starvation, we hypothesized that starvation not only increases the formation of autophagosomes but also increases their mobility in axons. Indeed, the frequency of autophagosome movement was increased by food restriction (Fig. 10*D*). To further confirm this hypothesis, we cultured primary motor neurons from the *dnc-1(RNAi)* worms in serum-depleted medium, and quantified the mobility of autophagosomes by monitoring the movement of DsRed-tagged Lgg1 in axons. As we expected, starvation significantly increased the speed and run-length of moving Lgg1 puncta, especially the retrograde run-length, in the *dnc-1(RNAi)* worms (p<0.0001) (Fig. 10*E, F*). Conversely, neurons treated with rapamycin showed no detectable change in the transport of autophagosomes (Fig. 10*E, F*). Histograms showing the distribution of the velocity and distance of autophagosome movement also demonstrated a significant increase of fast- and long-moving vesicles in the starved cells, especially in retrograde transport (Fig. 10*G, H*). For example, the percentage of vesicles that moved more than 8 μm retrogradely increased from 6.9% (*dnc-1(RNAi)* control) to 27.0% (*dnc-1(RNAi)* starvation), whereas the change was only from 12.7% to 19.7% in the anterograde direction (Fig. 10*H*).

**Figure 11:**
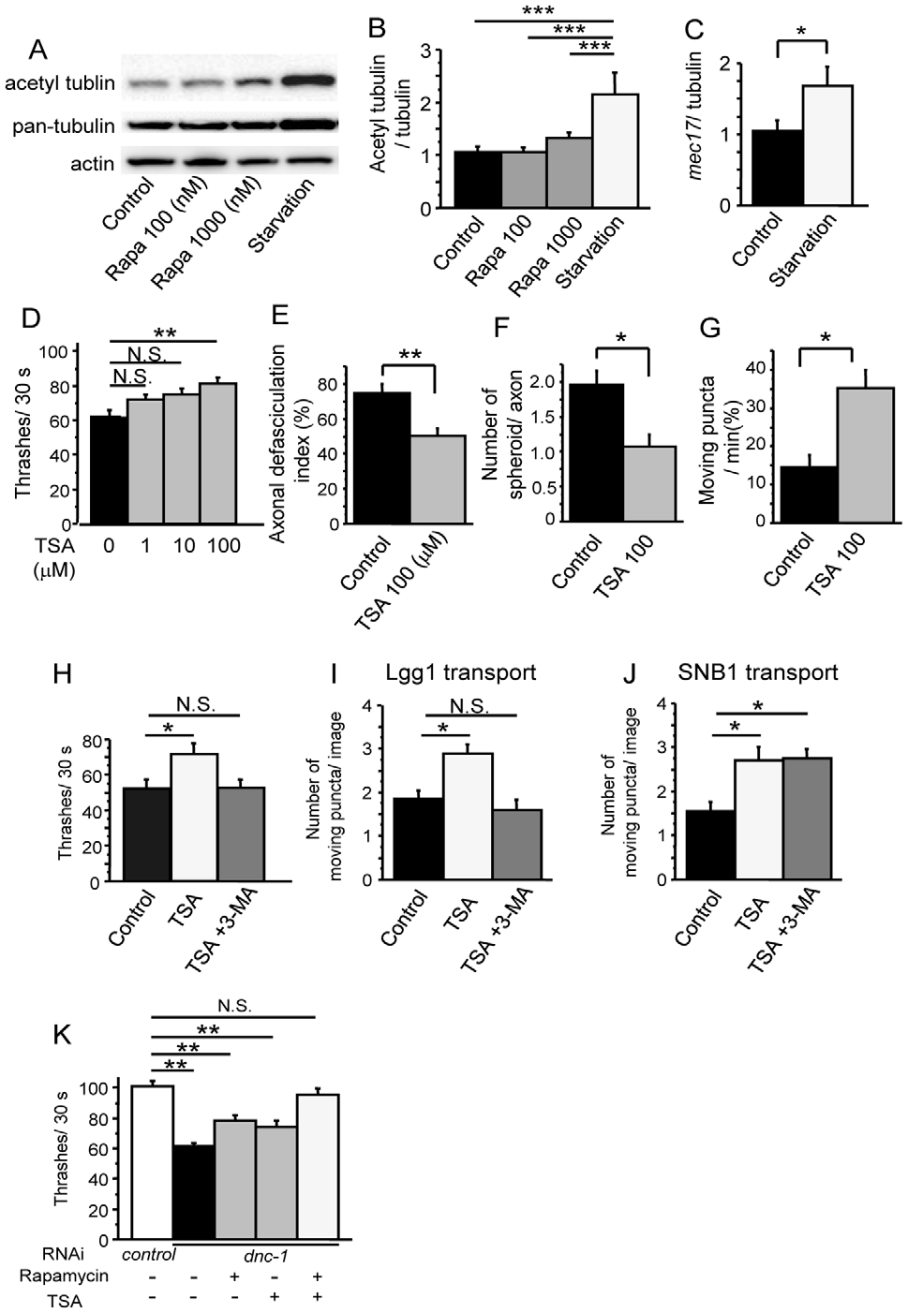
The effects of tubulin acetylation on the transport of autophagosome and neurodegneration in the *dnc-1* *(* *RNAi* *)* worms. (*A, B*) Immunoblots of primary cultured cells using antibodies against acetylated tubulin, pan-tubulin, and actin (n = 5). (*C*) The mRNA levels of *mec17* measured by real-time RT-PCR. The data shown are ratios to the mRNA levels of *tba1*, the gene encoding alpha-tubulin. (*D*) Effect of trichostatin A (TSA) on the locomotor function of the *dnc-1(RNAi)* worms (n = 35 for each group). (*E–G*) Effect of TSA (100 μM) on the axonal degeneration of the *dnc-1(RNAi)* worms (*E, F*) and on autophagosome mobility (*G*) (n = 15 for each group). (*H*) The inhibition of autophagy by 3-MA (10 mM) negates the effect of TSA treatment on the motor function of the *dnc-1(RNAi)* worms (n = 35 for each group). (*I, J*) The number of moving puncta (*I*, Lgg1; *J*, SNB1) was counted using kymographs derived from *in vivo* time-lapse images (n = 20 images for each analysis). Treatment with 3-MA negates the effect of TSA treatment on the transport of Lgg1 (*I*), but not the transport of SNB1 (*J*). (*K*) Combination therapy of rapamycin (100 μM) and TSA (100 μM) has synergistic effects on the locomotive functions of the *dnc-1(RNAi)* worms (n = 35 for each group). Statistical analyses were performed by one-way ANOVA followed by the Bonferroni/Dunn post hoc test for (*B*), Dunnett's post hoc test (*D, H–K*), and Student's t test (*C, E–G*) (*p<0.05, **p<0.001, and ***p<0.0001). The error bars are S.E.M.

Finally, we investigated how starvation stimulates the axonal transport of autophagosomes and assessed whether drugs that mimic the molecular mechanisms of starvation enhanced its effect. The acetylation of tubulin is known to stabilize microtubules and activate axonal transport by the subsequent recruitment of the molecular motors kinesin-1 and dynein/dynactin to microtubules [46], [47]. Therefore, we assessed the acetylation state of alpha-tubulin in our cultured cell assay. Starvation increased the levels of acetylated tubulin, but this effect was not detected in cells treated with rapamycin (Fig. 11*A, B*). Moreover, real-time quantitative PCR demonstrated that starvation, but not rapamycin, significantly increased the mRNA levels of *mec-17*, an enzyme that acetylates tubulin in *C. elegans* [48] (Fig. 11*C*). Taken together, our results suggest the possibility that starvation mitigated axonal degeneration by activating autophagy and promoting the axonal transport of autophagosomes via the acetylation of tubulin in the *dnc-1(RNAi)* worms. To test this hypothesis, we examined the effects of TSA, an HDAC inhibitor that facilitates tubulin acetylation, on the phenotypes of the *dnc-1(RNAi)* worms. Although treatment with TSA did not exhibit substantial effects on the phenotype of the *control(RNAi)* worms (Fig. S4 *A–C*), this treatment showed a significant effect on the locomotory function of the *dnc-1(RNAi)* worms in a dose-dependent manner, and attenuated the axonal degeneration without alteration of *dnc-1* knockdown efficiency (Fig. 11*D–F*, Fig. S3 *A, B, and D*). As expected, TSA increased the mobility of autophagosomes (Fig. 11*G*). Interestingly, treatment with 3-MA dampened the effect of TSA on locomotion (Fig. 11*H*). On the contrary, the worms treated with both TSA and 3-MA showed decreased transport of autophagosomes without defects in the transport of synaptobrevin (Fig. 11*I, J*). Furthermore, we also examined the effect of combination therapy with rapamycin and TSA. Although treatment with rapamycin or TSA alone had limited effects in comparison with *control(RNAi)* worms, the combination of rapamycin and TSA had greater effects such that locomotion was restored in the worms treated with these two drugs to the levels observed in the *control(RNAi)* worms (Fig. 11*K*).

## Discussion

In the present study, we generated a novel *C. elegans* model that mimics the down-regulation of dynactin 1 observed in the motor neurons of SALS patients. Using this model, we investigated whether the quantitative loss of DNC-1/dynactin 1 causes motor neuron degeneration. Our results showed that the knockdown of *dnc-1* caused progressive motor deficits in *C. elegans*, and the pathological changes observed in this model shared several features with those seen in SALS patients, e.g., the axonal accumulation of membranous structures, such as mitochondria and autophagosomes, and motor neuron degeneration characterized by axonal degeneration including axonal spheroids. We also observed the disrupted transport of autophagosomes in the degenerated motor neurons of this model. Interestingly, our model exhibited adult-onset motor neuron degeneration even though the *shRNA::gfp* had already expressed in the larval stage. Given that the patients carrying mutant *DCTN1* and SALS patients exhibit an adult-onset motor neuron degeneration, it is possible that developing motor neurons are resistant to the disruption of DNC-1/dynactin 1. However, differentiated motor neurons may be vulnerable to the detrimental effects of dynactin 1 depletion, since they require more efficient transport system to maintain axonal homeostasis than developing neurons. Together, these findings indicate that this *dnc-1*-KD *C. elegans* model is a powerful tool for understanding the relationship between the disrupted transport of autophagosomes, neurodegeneration, and motor phenotype.

The mechanism of autophagosome accumulation in motor neurons harboring a motor protein abnormality was shown directly by our analysis of autophagosomal transport; namely, the knockdown of *dnc-1* decreased the transport of autophagosomes and shortened their run-length. Physiological cargoes typically use multiple motors, and their run-lengths are correlated with the number of coordinated motor proteins [49]. Our results showed that the knockdown of *dnc-1* reduced the speed and distance of retrograde transport by approximately half. These results are consistent with previous *in vitro* studies of dynein showing that the run-length of retrograde motor complexes is reduced by approximately half in cells lacking dynactin 1 [49], [50]. Our data indicated that the knockdown of *dnc-1* also affected the anterograde transport of autophagosomes, which is consistent with previous reports showing that a defect in retrograde transport led to dysregulated movements in both directions [51], [52].

The relationship between the decreased DNC-1/dynactin 1 levels, the increased number of autophagosomes, and axonal degeneration was confirmed by our observations that the *dnc-1(RNAi)* worms showed an abnormal accumulation of autophagosomes and that their locomotory defects and axonal degeneration were correlated with the accumulation of autophagosomes. Furthermore, the *control(RNAi)* worms treated with 3-MA, an inhibitor of autophagy, showed the same phenotype as the *dnc-1(RNAi)* worms, including defective locomotory function and degenerated axons. Taken together, our findings in the *dnc-1(RNAi) C. elegans* model provide direct evidence that the lack of DNC-1/dynactin 1 in dynein/dynactin motor complexes leads to slow, short-distance movements of autophagosomes, followed by their axonal accumulation, and neurodegeneration.

It is clinically important to determine whether the activation of autophagy could be an effective therapeutic strategy against neurodegenerative diseases, especially when the transport of autophagosomes is disrupted. In previous studies, the effects of rapamycin, which induces autophagosome formation [9], against models of neurodegeneration were controversial [53]–[55]. In the present study, rapamycin only slightly ameliorated the motor dysfunction of the *dnc-1(RNAi)* worms, although its effects were substantially enhanced by the addition of TSA which enhances the acetylation of tubulin. Given that tubulin acetylation was shown to stimulate axonal transport [47], our results suggest that combination therapy with rapamycin and TSA, attenuated the neurodegeneration and locomotory dysfunction of this model by facilitating the formation and axonal transport of autophagosomes.

Although it is still possible that the disrupted transport of other organelles such as mitochondria are also involved in the pathogenesis of motor neuron degeneration in the *dnc-1(RNAi)* worms, the observation that 3-MA, an inhibitor of autophagy, almost completely abrogated the benefit effects of TSA suggests a substantial role for autophagosomal transport in the functional maintenance of motor neurons. This view is further supported by the fact that the worms treated by both TSA and 3-MA showed the decreased transport of autophagosomes without defects in the transport of synaptobrevin.

In conclusion, we found that decreased levels of dynactin 1 in motor neurons induce neurodegeneration at least partially via the disruption of the axonal transport of autophagosomes. The therapeutic strategy we examined in this study could be expanded to other neurodegenerative disorders, since the accumulation of autophagosomes and disrupted axonal transport are common features of many neurodegenerative diseases. Future study is needed to explore the effectiveness and safety of the treatments that stimulate the transport of autophagosomes in the mammalian central nervous system.

## Supporting Information

Figure S1 **Expression pattern of** ***shRNA*** **::GFP and morphology of ventral motor neurons during embryonic and larval stage.** (*A, B*) Representative confocal micro scopic image of *shRNA*::GFP expression during embryonic stages. GFP was not observed in the eggs even after delivery (asterisks in *A, B*). (*C–E*) GFP expression were observed in the ventral motor neurons (black arrows in *C*) from L1 (larval 1) stage of the worms. The ventral nerve axons (white arrows in *D, E*) did not exhibit abnormal changes such as axonal swellings or defasciculations during L1-4. Scale bars = 20 μm (*A–C*), 100 μm (low magnification image in *D, E*), or 50 μm (high magnification in *D, E*).
(TIF)

Figure S2 **Expression pattern of the Lgg1::DsRed in the** ***control*** *(* ***RNAi*** *)* **worm and the** ***dnc-1*** *(* ***RNAi*** *)* **worm.** (*A, B*) Representative fluorescent microscopic views of the Lgg1::DsRed in the ventral nerve cord of *control(RNAi)* worms (*A*) and *dnc-1(RNAi)* worms (*B*). The Lgg1 puncta (asteriscs in *B*) was abundant in the *dnc-1(RNAi)* worms (*B*). (*C*) Co-localization of DsRed and GFP fluorescence in the axonal spheroids (arrows) indicating that the autophagosomes (asteriscs) were accumulated in the axonal spheroids in the *dnc-1(RNAi)* worms. Scale bar = 10 μm (*A–C*).
(TIF)

Figure S3 **Pharmacological treatment or starvation did not alter the efficiency of the** ***dnc-1*** **knock-down.** (*A–D*) The representative image of GFP and *in situ* hybridization against *dnc-1* of ventral cholinergic motor neurons in the *conrol(RNAi)* (*A*) and *dnc-1(RNAi)* (*B*, no treatment; *C*, treated with starvation; *D*, treated with TSA). Scale bars = 10 μm.
(TIF)

Figure S4 **Treatment with TSA did not alter the locomotor function or the axonal integrity of the** ***control*** *(* ***RNAi*** *)* **worms.** (*A*) Effect of trichostatin A (TSA) on the locomotor function of the *control(RNAi)* worms (n = 35 for each group). (*B, C*) Effect of TSA (100 μM) on the axonal degeneration of the *dnc-1(RNAi)* worms (n = 15 for each group). Statistical analyses were performed using Student's t test.
(TIF)

Movie S1 **Representative transport of SNB-1::DsRed puncta** (**red**) **in a ventral motor neurons from the control worm.**
(MPEG)

Movie S2 **Representative transport of SNB-1::DsRed puncta** (**red**) **in a ventral motor neurons from the** ***dnc-1*** **KD worm.**
(MPEG)

Movie S3 **Representative transport of Lgg1::DsRed puncta** (**red**) **in a primary motor neuron from the control worm.**
(MPEG)

Movie S4 **Representative transport of Lgg1::DsRed puncta** (**red**) **in a primary motor neuron from the** ***dnc-1*** **KD worm.**
(MPEG)

Movie S5 **Representatie transport of Lgg1::DsRed puncta** (**red**) **in a ventral motor neurons from the control worm.**
(MPEG)

Movie S6 **Representatie transport of Lgg1::DsRed puncta** (**red**) **in a ventral motor neurons from the** ***dnc-1*** **KD worm.**
(MPEG)

Materials and Methods S1 **Detailed materials and methods for** ***C. elegans*** **and human protocols.**
(DOCX)
