# Supplementary figures and images for: dnc-1/dynactin 1 Knockdown Disrupts Transport of Autophagosomes and Induces Motor Neuron Degeneration
Source: PLoS One. 2013 Feb 7;8(2):e54511. doi: 10.1371/journal.pone.0054511 (PMC3567092; doi:10.1371/journal.pone.0054511)

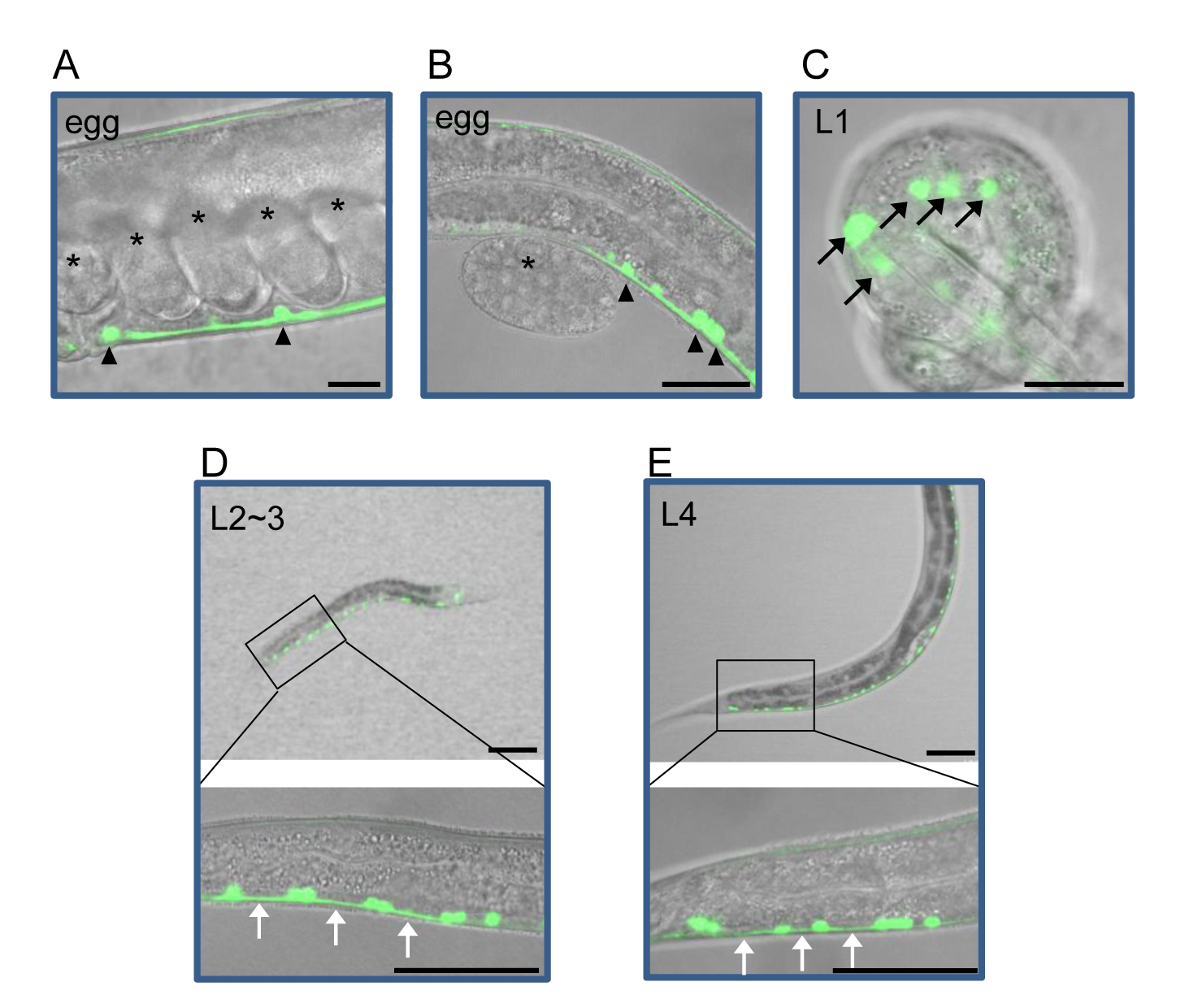

Supplement: Figure S1 — Expression pattern of shRNA ::GFP and morphology of ventral motor neurons during embryonic and larval stage. (A, B) Representative confocal micro scopic image of shRNA::GFP expression during embryonic stages. GFP was not observed in the eggs even after delivery (asterisks in A, B). (C–E) GFP expression were observed in the ventral motor neurons (black arrows in C) from L1 (larval 1) stage of the worms. The ventral nerve axons (white arrows in D, E) did not exhibit abnormal changes such as axonal swellings or defasciculations during L1-4. Scale bars = 20 μm (A–C), 100 μm (low magnification image in D, E), or 50 μm (high magnification in D, E). (TIF) [file pone.0054511.s001.tif]

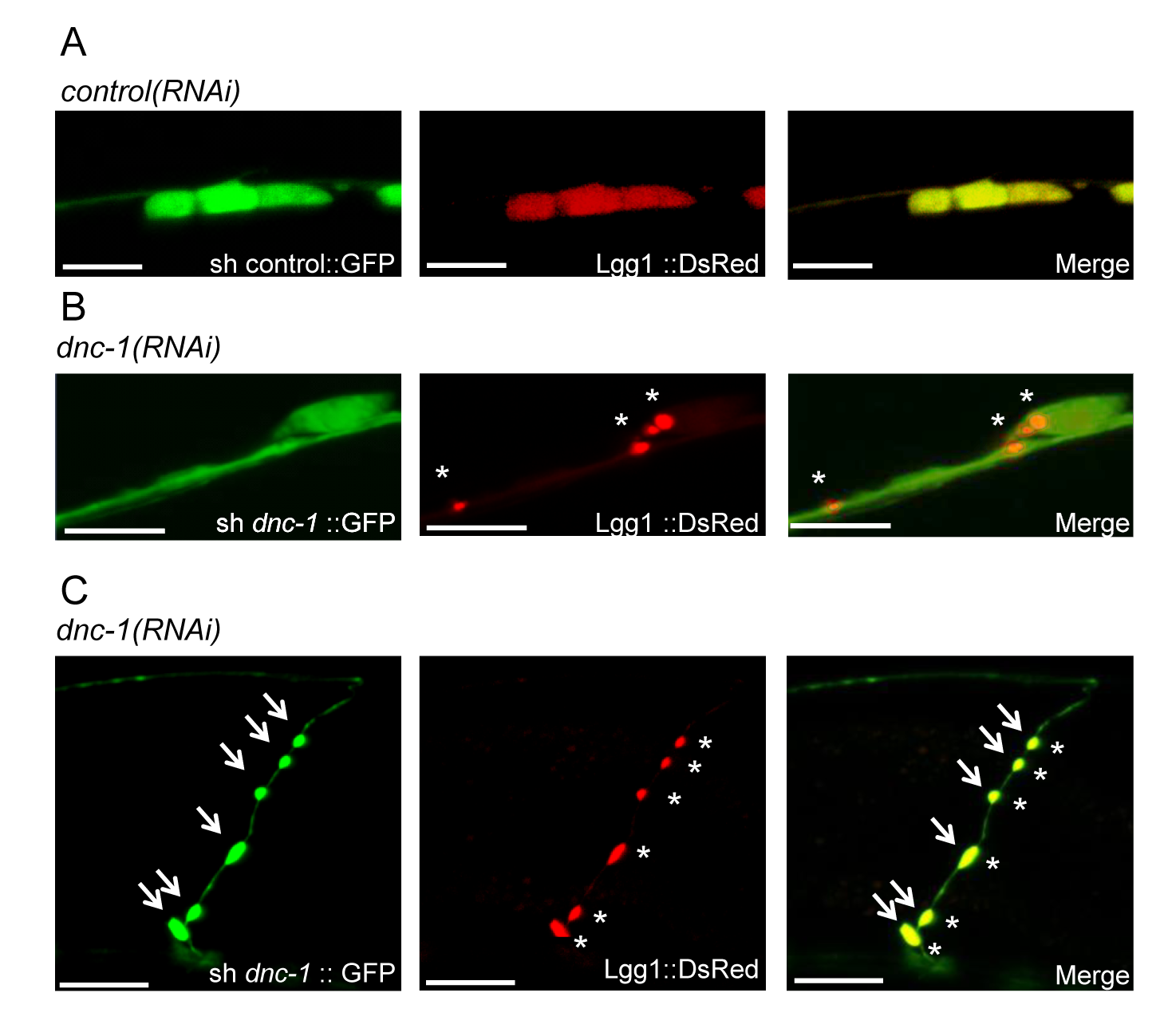

Supplement: Figure S2 — Expression pattern of the Lgg1::DsRed in the control ( RNAi ) worm and the dnc-1 ( RNAi ) worm. (A, B) Representative fluorescent microscopic views of the Lgg1::DsRed in the ventral nerve cord of control(RNAi) worms (A) and dnc-1(RNAi) worms (B). The Lgg1 puncta (asteriscs in B) was abundant in the dnc-1(RNAi) worms (B). (C) Co-localization of DsRed and GFP fluorescence in the axonal spheroids (arrows) indicating that the autophagosomes (asteriscs) were accumulated in the axonal spheroids in the dnc-1(RNAi) worms. Scale bar = 10 μm (A–C). (TIF) [file pone.0054511.s002.tif]

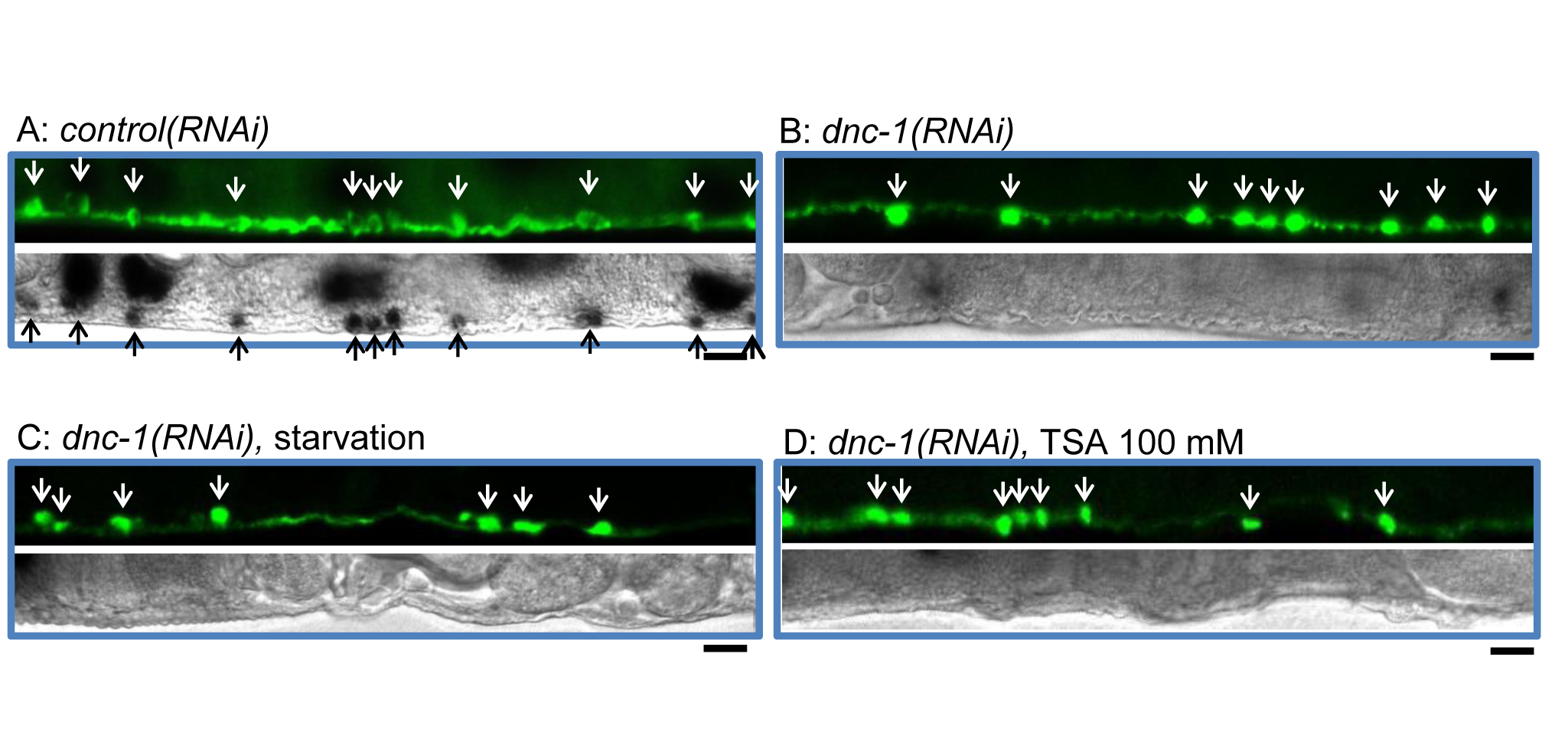

Supplement: Figure S3 — Pharmacological treatment or starvation did not alter the efficiency of the dnc-1 knock-down. (A–D) The representative image of GFP and in situ hybridization against dnc-1 of ventral cholinergic motor neurons in the conrol(RNAi) (A) and dnc-1(RNAi) (B, no treatment; C, treated with starvation; D, treated with TSA). Scale bars = 10 μm. (TIF) [file pone.0054511.s003.tif]

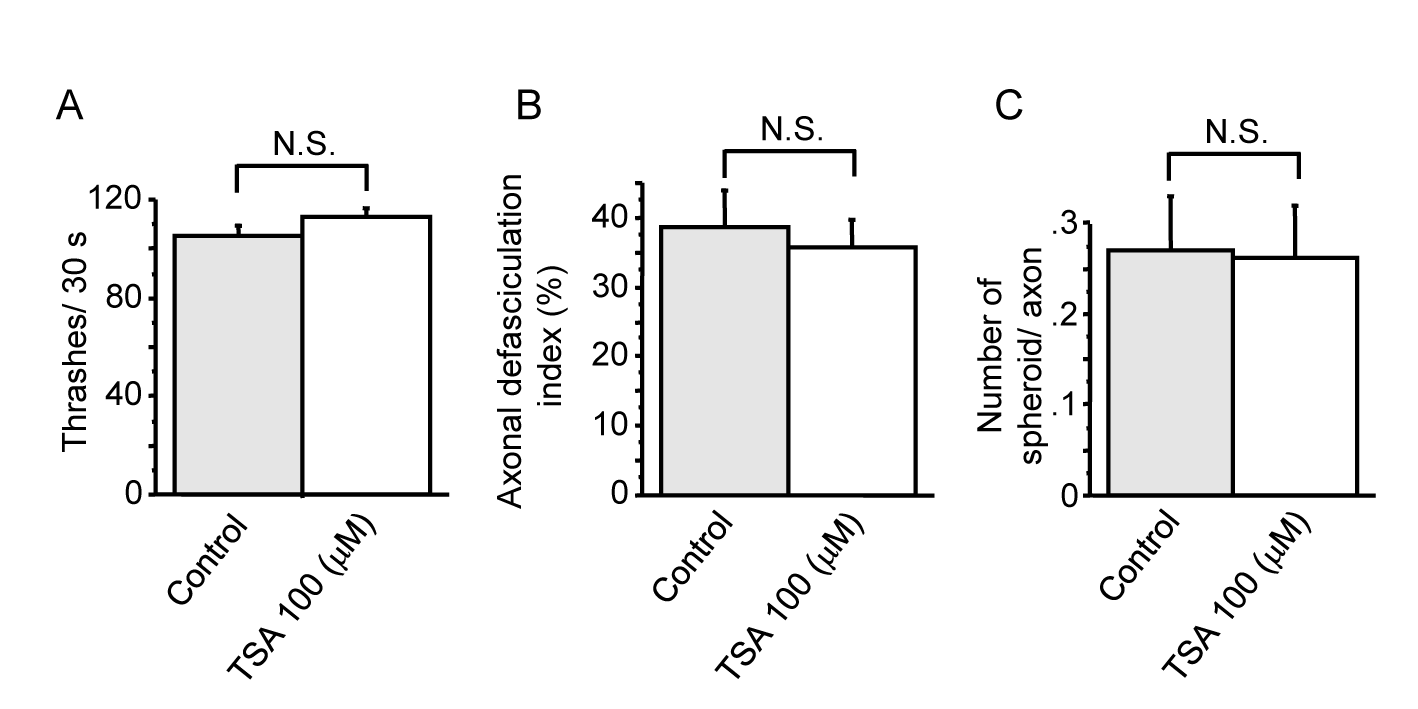

Supplement: Figure S4 — Treatment with TSA did not alter the locomotor function or the axonal integrity of the control ( RNAi ) worms. (A) Effect of trichostatin A (TSA) on the locomotor function of the control(RNAi) worms (n = 35 for each group). (B, C) Effect of TSA (100 μM) on the axonal degeneration of the dnc-1(RNAi) worms (n = 15 for each group). Statistical analyses were performed using Student's t test. (TIF) [file pone.0054511.s004.tif]
