# Supplementary material for: dnc-1/dynactin 1 Knockdown Disrupts Transport of Autophagosomes and Induces Motor Neuron Degeneration
Source: PLoS One. 2013 Feb 7;8(2):e54511. doi: 10.1371/journal.pone.0054511 (PMC3567092; doi:10.1371/journal.pone.0054511)
Supplement: Materials and Methods S1 — Detailed materials and methods for C. elegans and human protocols. (DOCX) [file pone.0054511.s011.docx]

**Supporting Materials and Methods**

***C. elegans* Protocols.** We used the Bristol strain (N2) as the wild-type animal. To generate the transgenic lines, DNA solutions (20 ng/μl) were co-injected into hermaphrodite gonads along with the marker gcy-8::GFP as described previously (Mello et al, 1991; Yu et al, 1997). We co-injected gcy::GFP together with the vector carrying *shRNA* for genotyping the transgenic worms. For quick and efficient selection of transgenic worms, we picked up the worms with gcy::GFP in the head (AFD) neurons, since gcy::GFP was brighter than acr2::GFP in ventral motor neurons and easily to distinguish under stereomicroscope. In most of the experiments, other than whole mount *in situ* hybridization of worms, we selected the worms expressing acr2::*shRNA*::GFP in more than thirty motor neurons. The person who performed this selection (Kaori Kawai) was blinded to the genotype of the animal and was different from the person who performed analysis (K.I.). Double transgenic worms were obtained by co-injecting two plasmids containing different fluorescent proteins. In the experiments that required large quantities of synchronized *C. elegans* or primary cultured neurons from *C. elegans*, we cultured the worms in liquid medium (S basal medium with concentrated OP50) as described previously (Stiernagle, 2006).

**Constructs and *C. elegans* Strains.** The shRNAs were designed based on the following sequences: scrambled (control) shRNA-set1, (5′-AACGGCGGATTGACCGTA-3′); *dnc-1 siRNA*-1(101), (5′-ATCACTCCAACCCAATCACC-3′); and *dnc-1*-*siRNA*-2 (2888), (5′-TACCCATCCTGCTTGCCAAG -3′). Each shRNA contained the loop structure, (5′-GTTTTGGCCACTGACTGAC-3′).

To generate an ENTRY vector, a BLOCK-iT Pol II miR RNAi expression vector kit with EmGFP (Invitrogen) was used according to the manufacturer’s instructions. These vectors were cloned into the pDONR 221 vector and then subcloned into the destination vector (Dest_acr2p). Dest_acr2p was designed by converting pOKU23_acr2p into a Gateway® destination vector. After injecting these constructs into the gonads, we generated the following C. elegans strains: SBG7, 8, and 15 N2; Ex[acr2 promoter::*dnc-1 (RNAi-1)::gfp*] and SBG20, 24, and 25 N2; Ex[acr-2 promotor*::dnc-1 (RNAi-2)::gfp*]. All six lines were used for the survival and body bend assays, and SBG8 was employed for further analysis.

The SNB-1 (GenBank accession number: NM_072287.3) and Lgg-1 genes (NM_062876.4), without stop codons, were cloned from a C. elegans cDNA library into the Gateway expression vector pENTR/D-TOPO and transferred to Dest-acr2p_DsRed (SNB-1::DsRed, Lgg-1::DsRed).

**Whole Mount *in situ* Hybridization.** Whole mount in situ hybridization was performed as described previously (Jiang *et al.*; 2007, Takada *et al.* , 2005). Briefly, adult worms were collected and fixed with PEM buffer (0.1 M PIPES, 1 mM MgSO4, 1 mM EGTA, pH 6.9). Fixed worms were treated with methanol and proteinase K to permeabilize the cuticle, and then prehybridized at 60°C for 1 h. A digoxigenin-labeled cRNA probe was generated from a linearized plasmid for *dnc-1* (NM_069632.5; nt 3525–3887; 363 bp) using SP6 or T7 polymerase (Roche Diagnostics, Basel, Switzerland). After prehybridization, the worms were hybridized with a digoxigenin-labeled cRNA probe overnight at 60°C. The washed worms were then incubated with an alkaline phosphatase-conjugated, anti-digoxigenin antibody (Roche Diagnostics). The signal was visualized with nitro blue tetrazolium/5-bromo-4-chloro-3-indolyl phosphate (Roche Diagnostics).

**Western Blot Analysis.** Western blot analyses were performed as described previously (Katsuno et al., 2003). Briefly, after being treated with rapamycin (100 or 1000 nM) or starved by serum depletion for 12 h, primary cultured cells from C. elegans embryos were lysed in RIPA buffer (Thermo Scientific, Rockford, IL, USA) containing a protease inhibitor mixture (Roche Diagnostics). Cell lysates were separated by SDS-PAGE (5–20% gradient gel) and analyzed by western blotting and ECL Plus detection reagents (GE Healthcare). The following primary antibodies were used: anti-alpha tubulin mouse monoclonal (1:10000; SIGMA-ALDRICH, St. Louis, MO, USA) and anti-acetylated tubulin mouse monoclonal (1:1000; SIGMA-ALDRICH).

**Quantitative real-time PCR**. Mec-17 mRNA levels were determined by real-time PCR as described previously (Ishigaki et al, 2002). Briefly, total RNA (1.6 μg each) from primary cultured cells treated with rapamycin, serum depletion, and control were reverse transcribed into first-strand cDNA using SuperScript III reverse transcriptase (Invitrogen). Real-time PCR was performed in a total volume of 25 μl, containing 12.5 μl of QuantiTect SYBR Green PCR Master Mix and 0.4 μM of each primer (Qiagen, Valencia, CA, USA), and the product was detected using the iCycler system (Bio-Rad Laboratories, Hercules, CA, USA). The reaction conditions were 95°C for 5 min and then 40 cycles of 10 s at 95°C followed by 30 s at 60°C. For an internal standard control, the expression level of alpha-tubulin (tba1) was quantified simultaneously. The following primers were used: 5′-TAATCCATCAGTCACTCTTCT-3′ and 5′-GGTATCAGTCATTCCAGTTC-3′ for mec17, and 5′-GTACACTCCACTGATCTCTGCTGACAAG-3′ and 5′-CTCTGTACAAGAGGCAAACAGCCATG-3′ for tba1. The threshold cycle of each gene was determined as the number of PCR cycles at which the increase in reporter fluorescence was 10 times that of the baseline signal. The weight of the gene contained in each sample was equal to the log of its starting quantity, and the standardized expression level in each worm was equal to the weight ratio of each gene to that of tba1.

**Electron microscopy.** A conventional two-step fixation method was performed as described previously (Hall et al, 1997), with a slight modification. Worms were anesthetized with 8% ethanol and then fixed in 2% glutaraldehyde and 2% paraformaldehyde in 0.1 M phosphate buffer (pH 7.4) for 2 h at 4°C. After two 10-min rinses in buffer, the samples were post-fixed in 1% osmium tetroxide in 0.1 M phosphate buffer for 1 h at 4°C in the dark, rinsed 3 times with buffer for a total of 30 min, dehydrated through an ethanol series, passed through propylene oxide, and then embedded in epoxy resin. Traverse, serial, ultra-thin sections through the body were collected on mesh grids and post-stained with uranyl acetate and lead citrate. The ventral cord neurons were examined on a JEM-1400EX electron microscope at 80 kV.

**Protocols for human samples**

***In situ* hybridization.** Frozen, 10-μm-thick spinal cord sections of autopsy material from SALS (44 years-old female, 63 years-old male, and 74 years-old male) and control patients with other neurodegenerative diseases (52 years–old male with pneumonia, 65 years-old male with brain infarction, and 78 years-old female with interstitial pneumonia) were prepared and immediately fixed in 4% paraformaldehyde. The sections were then treated twice with 0.1% diethylpyrocarbonate for 15 min and prehybridized at 45°C for 1 h. A digoxigenin-labeled cRNA probe was generated from a linearized plasmid for dynactin 1 (*DCTN-1*; NM_004082; nt 2392–2774; 383 bp) using SP6 or T7 polymerase (Roche Diagnostics). The sections were hybridized with the digoxigenin-labeled cRNA probe overnight at 45°C. The washed sections were incubated with an alkaline phosphatase-conjugated anti-digoxigenin antibody (Roche Diagnostics), and the signal was visualized with nitro blue tetrazolium/5-bromo-4-chloro-3-indolyl phosphate (Roche Diagnostics). No hybridization signal was observed with the sense probe in spinal motor neurons. To investigate the correlation between expression levels of *DCTN-1* and LC3 in an individual motor neuron we used consecutive transverse spinal cord sections. Ten sets of consecutive sections for the gene or protein of interest were prepared from each patient.

References

Hall DH, Gu G, Garcia-Anoveros J, Gong L, Chalfie M, Driscoll M (1997) Neuropathology of degenerative cell death in Caenorhabditis elegans. *J Neurosci* **17:** 1033-1045

Ishigaki S, Liang Y, Yamamoto M, Niwa J, Ando Y, Yoshihara T, Takeuchi H, Doyu M, Sobue G (2002) X-Linked inhibitor of apoptosis protein is involved in mutant SOD1-mediated neuronal degeneration. *J Neurochem* **82:** 576-584

Mello CC, Kramer JM, Stinchcomb D, Ambros V (1991) Efficient gene transfer in C.elegans: extrachromosomal maintenance and integration of transforming sequences. *EMBO J* **10:** 3959-3970

Stiernagle T (2006) Maintenance of C. elegans. *WormBook***:** 1-11

Yu S, Avery L, Baude E, Garbers DL (1997) Guanylyl cyclase expression in specific sensory neurons: a new family of chemosensory receptors. *Proc Natl Acad Sci U S A* **94:** 3384-3387
